# A Hybrid Metaheuristic for High-Dimensional Constrained Optimization: Applications to Logistics and UAV Path Planning

**DOI:** 10.3390/biomimetics11060361

**Published:** 2026-05-22

**Authors:** Yarong Li, Chuandong Qin

**Affiliations:** 1 School of Mathematics and Information Science, North Minzu University, Yinchuan 750021, China; 2 Ningxia Key Laboratory of Intelligent Information and Big Data Processing, North Minzu University, Yinchuan 750021, China; 2012036@nmu.edu.cn

**Keywords:** swarm intelligence, metaheuristic optimization, Improved Pied Kingfisher Optimizer, multi-strategy integration, constrained optimization

## Abstract

Inspired by the hovering, diving, and cooperative hunting behaviors of the pied kingfisher, the Pied Kingfisher Optimizer (PKO) has demonstrated competitive performance in optimization tasks. However, it exhibits several phase-specific limitations, including uneven population distribution caused by random initialization, insufficient use of historical information during exploration, over-reliance on the global best during exploitation, and weakly guided perturbation in the symbiosis phase. To address these issues, this study proposes an Improved Pied Kingfisher Optimizer (IPKO), which incorporates biologically inspired adaptive strategies. Drawing inspiration from the kingfisher’s diverse perching, gaze adjustment during hovering, evasive diving after failed strikes, and territory shifting based on flock position, four mechanisms are developed. Specifically, sine chaotic opposition-based initialization enhances population diversity; adaptive directional search regulates the exploration–exploitation balance; stochastic perturbation-based information fusion improves the ability to escape local optima; and centroid-based adaptive boundary handling strengthens constraint adaptability. The performance of IPKO is evaluated on the CEC2017 benchmark suite (10, 30, 50, and 100 dimensions) and two real-world engineering problems. Experimental results show that IPKO achieves superior overall performance compared with eleven state-of-the-art algorithms, with statistical significance confirmed by the Friedman test and Holm’s post-hoc procedure. Ablation studies further verify the contribution of each strategy. In engineering applications such as cold chain logistics and dynamic multi-UAV cooperative path planning, the IPKO algorithm demonstrates superior solution quality, robustness, and constraint-handling capability compared with competing algorithms. These results demonstrate that IPKO is a robust and effective bio-inspired optimization approach for solving complex, high-dimensional constrained engineering problems.

## 1. Introduction

In nature, the pied kingfisher performs a remarkable sequence of behaviors—hovering to survey prey, diving to capture, and cooperating with conspecifics—to achieve efficient foraging in complex environments. This biological strategy offers a compelling metaphor for solving complex optimization problems in engineering. In the era of Industry 4.0 and smart manufacturing, engineering systems are generating increasingly complex optimization challenges that often involve high-dimensional, nonlinear, and multi-constrained decision spaces, making traditional deterministic methods inadequate. Metaheuristic optimization algorithms have become indispensable tools for solving complex optimization problems in science and engineering, particularly when traditional deterministic methods are ineffective due to nonlinearity, high dimensionality, and the absence of gradient information [1]. These algorithms have been successfully applied across a wide range of domains. In medicine, improved particle swarm optimization has been used to determine optimal mounting positions for medical robots, while enhanced whale optimization algorithms have been employed for COVID-19 detection [2,3]. In image processing, grey wolf optimizer variants have been applied to multilevel thresholding problems [4]. In power systems, metaheuristics have been widely used to solve optimal reactive power dispatch and economic emission dispatch problems [5,6]. In engineering optimization, various algorithms have demonstrated strong performance in solving complex design and planning problems [7]. These applications highlight the effectiveness and versatility of metaheuristic optimization methods. In addition to metaheuristic optimization methods, mathematical optimization approaches have also demonstrated strong capabilities in solving engineering optimization problems. For example, convex relaxation and convex modeling techniques have been successfully applied in hybrid AC/DC microgrid economic dispatch problems due to their computational efficiency and theoretical guarantees. Representative studies such as “Enabling high-efficiency economic dispatch of hybrid AC/DC networked microgrids: Steady-state convex bi-directional converter models” provide effective deterministic optimization frameworks for structured optimization problems [8].

Metaheuristic algorithms are generally classified into four categories: swarm intelligence (SI) algorithms, evolutionary algorithms (EA), physics-based algorithms, and human-based algorithms [9]. Swarm intelligence algorithms simulate collective behaviors of natural organisms, and include particle swarm optimization (PSO), grey wolf optimizer (GWO), whale optimization algorithm (WOA), Harris hawks optimization (HHO), marine predators algorithm (MPA), and golden jackal optimization (GJO) [10,11,12,13,14,15]. Evolutionary algorithms mimic biological evolution and natural selection, including genetic algorithms (GA) and differential evolution (DE) [16,17]. Physics-based algorithms are inspired by physical laws, such as the gravitational search algorithm (GSA) and simulated annealing (SA) [18,19]. Human-based algorithms model human learning or social behaviors, such as teaching–learning-based optimization (TLBO) [20]. According to the No Free Lunch theorem [21], no single optimization algorithm can achieve optimal performance across all problem domains. Therefore, continuous development and improvement of metaheuristic algorithms remain essential.

Despite their success, many metaheuristic algorithms still suffer from issues such as premature convergence, insufficient exploration in high-dimensional spaces, and weak adaptability under complex constraints [22]. To address these limitations, recent studies have increasingly focused on integrating multiple strategies within a unified optimization framework to enhance robustness and generalization capability. For example, adaptive and multi-strategy particle swarm optimization variants have been proposed to balance exploration and exploitation more effectively [23,24,25]. Similarly, algorithms such as GJO, MPA, and snake optimizer (SO) [26] have been widely improved through various enhancement strategies, such as opposition-based learning, chaotic mapping, adaptive perturbation, and hybrid update mechanisms, which has become a common approach to improving convergence accuracy and maintaining population diversity in metaheuristic optimization. These developments indicate that hybridization, adaptive mechanisms, and multi-strategy integration have become dominant trends in recent metaheuristic design, particularly for addressing high-dimensional and constrained optimization problems.

The Pied Kingfisher Optimizer (PKO) is a recently proposed swarm-based metaheuristic inspired by the hunting behavior of pied kingfishers [27]. In nature, a kingfisher first hovers to survey prey distribution (exploration), then dives sharply toward a selected target (exploitation), and occasionally performs cooperative fishing with conspecifics (symbiosis). Accordingly, PKO organizes its search into four phases: initialization (positioning of virtual kingfishers), exploration (global search via hovering simulation), exploitation (local search via diving), and symbiosis (information sharing among individuals). This biomimetic mapping provides an intuitive optimization framework. PKO has demonstrated competitive performance on benchmark functions and engineering applications, indicating its potential as an effective optimization method. However, like many swarm-based algorithms, PKO exhibits several limitations when dealing with complex optimization problems. First, its random initialization strategy may lead to insufficient population diversity and uneven spatial distribution, limiting early-stage exploration. Second, the exploration phase relies heavily on stochastic parameter switching without effectively utilizing historical information, resulting in reduced search efficiency. Third, the exploitation phase is strongly guided by the current global best solution, which may cause the algorithm to become trapped in local optima and suffer from premature convergence. Finally, the symbiosis phase employs random perturbations without directional guidance, leading to unstable performance, especially in high-dimensional or constrained scenarios. Although several studies have applied PKO to different problems, its phase-specific limitations remain largely unaddressed. These limitations collectively restrict the convergence accuracy, robustness, and scalability of the original PKO.

Motivated by the above observations, this paper proposes an Improved Pied Kingfisher Optimizer (IPKO) that further draws biological inspiration from previously unmodeled kingfisher behaviors. Specifically:

- Diverse perching (sine chaotic opposition-based initialization): In nature, kingfishers rarely perch at the same spot repeatedly; they survey from multiple perches to gain different perspectives. We simulate this by using sine chaotic mapping to generate diverse initial positions, then apply opposition-based learning to expand the search space, mimicking the bird’s ability to consider opposite directions.
- Gaze adjustment during hovering (adaptive directional search): While hovering, a kingfisher continuously adjusts its gaze based on both the best-known prey location and random movements of nearby birds. Our adaptive directional search guides exploration using both global best information and randomly selected individuals.
- Evasive diving after failed strikes (stochastic perturbation-based information fusion): When a kingfisher fails to catch prey, it performs an erratic dive to relocate. Similarly, our stochastic perturbation strategy adds controlled randomness to help the algorithm escape local optima.
- Territory shifting based on flock position (centroid-based adaptive boundary handling): A kingfisher’s territory is not rigid; it adapts based on the average position of the flock. We use the population centroid to dynamically adjust out-of-bound individuals, maintaining feasibility while preserving diversity.

The effectiveness of the proposed IPKO is evaluated on the IEEE CEC2017 benchmark suite with multiple dimensional settings (10, 30, 50, and 100). In addition, two real-world engineering problems, namely cold chain logistics routing and dynamic multi-UAV cooperative path planning, are considered to further validate its practical applicability. The performance of IPKO is compared with eleven state-of-the-art algorithms, including GA, PSO, DE, GWO, WOA, HHO, GBO, HBA, and the original PKO. Statistical significance is assessed using the Wilcoxon signed-rank test and Friedman test. Furthermore, ablation studies and parameter sensitivity analysis are conducted to verify the contribution and robustness of each proposed strategy.

The main contributions of this paper are summarized as follows:

1. A biomimetic enhancement framework that models four specific kingfisher behaviors (diverse perching, gaze adjustment, evasive diving, and territory shifting) into mathematical optimization strategies, extending the biological fidelity of the original PKO.
2. A sine chaotic mapping combined with opposition-based learning strategy is introduced to enhance population diversity and improve early-stage exploration.
3. An adaptive directional search mechanism is proposed to improve exploration efficiency and accelerate convergence.
4. A stochastic perturbation-based information fusion strategy is developed to prevent premature convergence and enhance the ability to escape local optima.
5. A centroid-based adaptive boundary handling mechanism is designed to effectively maintain feasibility under complex constraints.
6. Comprehensive experiments on benchmark functions and real-world engineering problems demonstrate that the proposed IPKO achieves superior performance in terms of convergence accuracy, robustness, and solution quality.

Based on these enhancements, this study provides a complete biomimetic optimization framework that not only faithfully models the pied kingfisher’s hunting behaviors but also demonstrates its practical transferability to two representative engineering systems: cold chain logistics (analogous to efficient prey routing) and dynamic multi-UAV cooperative path planning (analogous to flock hunting). The results confirm that biologically faithful algorithm design can yield superior performance in real-world optimization tasks.

The remainder of this paper is organized as follows. Section 2 introduces the original PKO and analyzes its limitations. Section 3 presents the proposed IPKO and its improvement strategies. Section 4 reports experimental results on benchmark functions. Section 5 discusses applications to real-world engineering problems. Finally, Section 6 concludes the paper and outlines future research directions.

## 2. Original Pied Kingfisher Optimization Algorithm (PKO)

The Pied Kingfisher Optimization (PKO) algorithm is a metaheuristic optimization method designed for solving single-objective optimization problems. The algorithm is constructed based on four search operators, namely perching, hovering, diving, and commensalism. These operators are mapped to four sequential phases: initialization, exploration, exploitation, and symbiosis.

### 2.1. Initialization Phase

PKO generates an initial population consisting of *N* candidate solutions randomly distributed within the feasible search space. The position of the *i*-th individual in the *j*-th dimension is defined as:

(1) $$X_{i,j}=LB+\left(UB-LB\right)\times \mathrm{rand},1\leq i\leq N,1\leq j\leq Dim$$

where rand∈[0,1] is a uniformly distributed random number, and LB and UB represent the lower and upper bounds of the decision space, respectively. This initialization strategy ensures feasibility but does not explicitly promote population diversity or spatial coverage.

### 2.2. Exploration Phase

The exploration phase is responsible for global search and is implemented through two complementary update strategies. The position update rule is given by:

(2) $$X_{new}\left(i,:\right)=X\left(i,:\right)+\alpha \cdot T\cdot (X\left(j,:)-X\left(i,:\right)\right),\left(i\neq j\right)$$

where α is a random vector sampled from a normal distribution, and *T* is a time-varying control parameter that determines the search mode. The parameter *T* switches between two update schemes with different step characteristics, facilitating variation in search range. One scheme produces relatively small perturbations, while the other allows larger position adjustments, thereby enhancing population dispersion.

### 2.3. Exploitation Phase

The exploitation phase focuses on refining candidate solutions in promising regions of the search space. The update rule is defined as:

(3) $$X_{\mathrm{new}}\left(i,:\right)=X\left(i,:\right)+HuntingAbility\times o\times \alpha \cdot \left(b-bestPosition\right)$$

where HuntingAbility is a scaling factor related to the fitness of individuals, *o* is a decreasing coefficient controlling the step size over iterations, α is a stochastic vector, and bestPosition denotes the current global best solution. This mechanism intensifies the search around high-quality solutions but introduces a strong dependency on the accuracy of the global best.

### 2.4. Symbiosis Phase

The symbiosis phase introduces stochastic interactions among individuals to improve population diversity and support escape from local optima. The update rule is expressed as:

(4) $$X_{\mathrm{new}}\left(i,:\right)=X\left(r,:\right)+o\times \alpha \cdot \left|X(i,:)-X(r,:)\right|$$

where *r* is a randomly selected individual index within the population. This operation perturbs the current solution using the relative distance between individuals. The activation of this phase is controlled by the Predation Efficiency (PE), which decreases linearly during the optimization process, thereby reducing randomness in later stages.

### 2.5. Summary and Motivation for Improvement

Although PKO provides a structured framework that integrates exploration and exploitation, several inherent limitations can be identified in its core components. These limitations directly motivate the design of the proposed Improved Pied Kingfisher Optimizer (IPKO).

- Initialization limitation:

The population is generated using a purely random initialization strategy, which does not guarantee uniform distribution or sufficient diversity. This may result in clustered individuals and inadequate coverage of the search space, particularly in high-dimensional problems.

This limitation motivates the introduction of a diversity-enhanced initialization strategy.

- Exploration limitation:

The exploration phase primarily depends on stochastic parameter sampling without incorporating any historical or population-level guidance. As a result, the search process may suffer from low efficiency, redundant exploration, and slow convergence.

This limitation motivates the development of a guided exploration mechanism that leverages population information.

- Exploitation limitation:

The exploitation phase relies heavily on the current global best solution to guide the search. When the global best is trapped in a local optimum, the population tends to converge prematurely, reducing solution quality.

This limitation motivates the design of an adaptive exploitation strategy to alleviate premature convergence.

- Symbiosis limitation:

The symbiosis phase introduces random perturbations without directional or adaptive control. This leads to unstable performance, as the mechanism may either fail to escape local optima or disrupt promising solutions.

This limitation motivates the incorporation of a controlled and adaptive local escape mechanism.

- Overall motivation:

To address the above limitations, this study proposes an Improved Pied Kingfisher Optimizer (IPKO), which enhances population diversity, improves search guidance, strengthens exploitation robustness, and introduces adaptive escape strategies. The details of these improvements are presented in Section 3.

## 3. Improved Pied Kingfisher Optimization Algorithm (IPKO)

### 3.1. Design Rationale of IPKO

Although the original Pied Kingfisher Optimizer (PKO) provides a biologically inspired framework that combines exploration and exploitation behaviors, its search dynamics still exhibit several limitations when solving high-dimensional constrained optimization problems. As analyzed in Section 2, these limitations mainly include: (i) insufficient population diversity during initialization, (ii) weak utilization of directional and historical information during exploration, (iii) premature convergence caused by excessive dependence on the global best solution during exploitation, and (iv) unstable boundary adaptation and local escape capability in constrained search spaces.

From a biomimetic perspective, pied kingfishers exhibit highly adaptive hunting behaviors in complex natural environments. During hovering, kingfishers continuously adjust gaze orientation to track moving prey and optimize attack direction. When prey capture fails, they perform evasive diving and dynamic repositioning to avoid repeated ineffective attacks. In addition, flock-level territory adjustment enables individuals to maintain spatial adaptability under environmental constraints. These adaptive biological behaviors provide important inspiration for improving the search dynamics of PKO.

Motivated by these observations, this study proposes an Improved Pied Kingfisher Optimizer (IPKO) based on a coordinated adaptive search framework. The proposed framework establishes behavior-oriented cooperation among different search mechanisms across various optimization stages. Specifically, four complementary mechanisms are developed: (1) a sine chaotic opposition-based initialization strategy inspired by diversified perch selection behavior to improve population distribution and spatial coverage; (2) an adaptive directional search mechanism inspired by hovering gaze adjustment behavior to enhance directional exploration and adaptive exploitation; (3) a stochastic binary perturbation-based information fusion strategy inspired by evasive diving behavior after failed predation to improve local-optimum escape capability; (4) a centroid-based adaptive boundary handling mechanism inspired by territory relocation and cooperative flock positioning behavior to strengthen feasible-region adaptability under boundary constraints.

**Figure 1:**
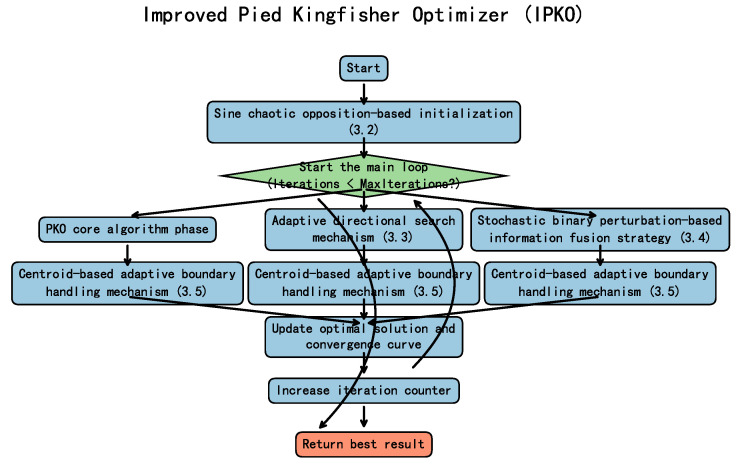
Flowchart of the IPKO Algorithm.

The proposed IPKO constructs a coordinated adaptive enhancement framework in which each mechanism targets a specific deficiency of PKO while cooperatively improving population diversity, search guidance, exploitation robustness, and constrained search adaptability. The overall framework of IPKO is illustrated in Figure 1, where 3.2–3.5 in the figure correspond to Section 3.2, Section 3.3, Section 3.4 and Section 3.5 respectively.

### 3.2. Sine Chaotic Opposition-Based Initialization Strategy

To overcome the limitations of purely random initialization, a sine chaotic opposition-based initialization strategy is introduced [28,29,30]. This strategy replaces random sampling with a structured population generation mechanism to improve spatial distribution and diversity.

First, a sine chaotic sequence is generated as:

(5) sine(i,j)=sin(π·sine(i,j−1)),j=2,…,dim

where sine(i,1) is initialized randomly in [0,1]. The sequence is then mapped to the search space:

(6) X(i,:)=lb+sine(i,:)·(ub−lb)

To further enhance diversity, an opposition-based learning strategy is incorporated:

(7) $$X^{op}\left(i,:\right)=r\cdot \left(ub-lb\right)-X\left(i,:\right)$$

where r∈[0,1] is a random scalar. Boundary constraints are enforced as:

(8) $$X^{op}\left(i,:\right)=\max\left(X^{op}\left(i,:\right),lb\right),\;X^{op}\left(i,:\right)=\min\left(X^{op}\left(i,:\right),ub\right)$$

The original and opposite populations are merged and ranked:

(9) $$X^{all}=\left[X;X^{op}\right]$$

and the best *N* individuals are retained:

(10) $$pop=X^{all}\left(index\left(1:N\right),:\right)$$

Compared with the purely random initialization in the original PKO, the proposed sine chaotic opposition-based strategy introduces a structured population generation mechanism. By leveraging the ergodicity of sine chaotic mapping and the symmetry of opposition-based learning, it produces an initial population with improved spatial coverage and reduced clustering. This leads to enhanced exploration capability in early iterations and faster convergence toward promising regions, providing a more reliable foundation for subsequent adaptive search processes, particularly in high-dimensional and multimodal optimization problems.

### 3.3. Adaptive Directional Search Mechanism

To overcome the insufficient guidance and low search efficiency of the original PKO during the exploration stage, an adaptive directional search mechanism is proposed. This mechanism is inspired by the hovering and visual tracking behaviors of pied kingfishers, as well as the environmental perception mechanism in crayfish optimization [31,32]. In natural hunting processes, pied kingfishers continuously adjust their hovering posture and gaze orientation according to prey movement and environmental feedback. By integrating global guidance, peer-based directional perception, adaptive intensity regulation, and stochastic angular perturbation, the proposed mechanism establishes a direction-aware neighborhood search framework.

The adaptive directional search is activated when

(11) r≤(1−PE)

where PE denotes the predation efficiency and r∈[0,1] is a uniformly distributed random number. A low predation efficiency indicates that the current search fails to approach promising regions, thereby triggering enhanced directional exploration.

The update procedure consists of the following five steps, each fully defined for reproducibility.

Step 1: Peer Individual Selection

A random individual is selected from the population to provide auxiliary directional information:

(12) $$r_{1}=randi\left(\left[1,\mathrm{Popsize}\right]\right)$$

This peer-based interaction reduces over-dependence on the global best solution and improves population-level directional adaptability.

Step 2: Global Direction Construction

A directional vector toward the current global best solution is constructed as:

(13) $$X_{2}=\left(\mathrm{Best}\_\mathrm{position}-X\left(i,:\right)\right)\times r$$

where r∈[0,1] is the same random number used in the activation condition. This vector represents the primary search tendency toward promising regions.

Step 3: Adaptive Search Intensity Regulation (*B*)

An adaptive modulation coefficient is introduced to balance exploration and exploitation over iterations:

(14) $$B=c\times \cos\left(\frac{\pi }{2}\times \left(1-\frac{t}{\mathrm{Maxiteration}}\right)\right),\;c=2$$

Here, *t* is the current iteration and Maxiteration is the maximum number of iterations. *B* increases from 0 to 2 as the optimization progresses. In early iterations (*t* small, B≈0), the search step is nearly zero, preserving population diversity. In later iterations (*t* close to Maxiteration, B≈2), the directional movement is fully activated, enabling strong local exploitation around promising regions. The constant c=2 defines the upper bound of the modulation intensity.

Step 4: Stochastic Angular Perturbation (θ)

To simulate omnidirectional gaze adjustment, an angular perturbation mechanism is introduced:

(15) θ=RouletteWheelSelection(p),p=1,2,…,360

Each angle θ (in degrees) is assigned an equal probability 1/360. The roulette wheel selection is implemented as:

(16) θ=⌈360·u⌉,u∼U(0,1)

The angle determines the direction of the sine/cosine components in the position update. A full circle ($0^{\circ }$ to $360^{\circ }$) allows unbiased omnidirectional sampling. For example, $\theta =0^{\circ }$ makes the cosine term dominate (moving directly toward the peer individual), while $\theta =90^{\circ }$ makes the sine term dominate (lateral movement). This mechanism prevents repeated movement along fixed directions and significantly enhances spatial exploration diversity.

Step 5: Direction-Aware Position Update with Perturbation Factor (*V*)

The final position update is formulated as:

(17) $$\begin{array}{cc} X_{\mathrm{new}}\left(i,:\right)= & X_{2}+\left(X\left(r_{1},:\right)-X\left(i,:\right)\right)\times \cos\left(\theta \right)\times B\times V\times \mathrm{rand}\\ \; & +X\left(r_{1},:\right)\times \sin\left(\theta \right)\times B\times \mathrm{rand} \end{array}$$

where:

- rand∈[0,1] is a uniformly distributed random number regenerated each time the equation is evaluated.
- *V* is a discrete random variable representing the environmental perception intensity. Based on the crayfish-inspired environmental quality evaluation in the reference, *V* is independently sampled for each activation:
  (18) V=randi([2,5])(integeruniformlydrawnfrom{2,3,4,5})

**Figure 2:**
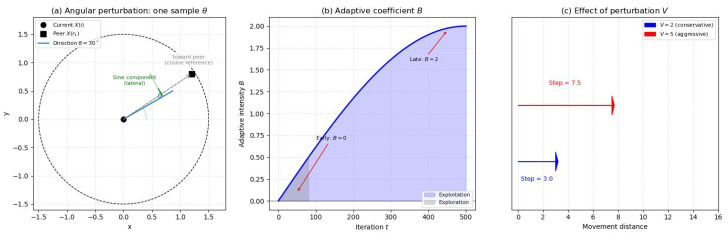
Schematic of the adaptive directional search parameters.

A smaller value (V=2 or 3) indicates that the current local environment is perceived as favorable, encouraging conservative directional contraction. A larger value (V=4 or 5) emulates poor environmental quality (e.g., oxygen deficiency or food scarcity), triggering a stronger perturbation to escape local optima. The stochastic nature of *V* introduces non-deterministic step-size bursts, further improving robustness. For illustrative purposes, Figure 2c presents two representative cases (V=2 and V=5), corresponding to conservative and aggressive perturbation behaviors, respectively.

The three parameters jointly determine the magnitude and direction of the search move:

- *B* provides an iteration-dependent global scaling that grows from 0 to 2, making early moves tentative and later moves become more directionally focused.
- θ decides how the movement is split into a radial component (toward the peer individual, via cosθ) and a lateral component (perpendicular to the peer direction, via sinθ).
- *V* randomly modulates the cosine-dominated radial step, amplifying it by a factor of 2 to 5. The sine term is not multiplied by *V*, preserving a baseline lateral exploration regardless of environmental perception.

Table 1 provides a concrete example for different parameter combinations under a fixed peer direction.

Figure 2 provides a schematic illustration to improve intuitive understanding:

- (a) Angular perturbation: A circle centered at the current individual X(i). The angle θ (sampled uniformly from $0^{\circ }$ to $360^{\circ }$) determines the search direction. The cosine component points toward the selected peer individual $X(r_{1})$, while the sine component is perpendicular.
- (b) Adaptive intensity *B* over iterations: A plot showing *B* increasing from 0 to 2 following a cosine curve, with early iterations (exploration) in gray and late iterations (exploitation) in blue.
- (c) Perturbation factor *V*: Two arrows representing step sizes for V=2 (short arrow) and V=5 (long arrow), illustrating how a larger *V* triggers a longer jump.

#### Comparison with the Original PKO

The original PKO relies primarily on stochastic movement without explicit directional coordination or adaptive modulation. In contrast, the proposed mechanism integrates:

- global guidance ($X_{2}$),
- peer-based directional perception ($X\left(r_{1},:\right)-X\left(i,:\right)$),
- iteration-dependent scaling (*B*),
- unbiased omnidirectional angle sampling (θ),
- stochastic step-size modulation (*V*).

This design yields a balanced exploration–exploitation transition, stronger convergence, and improved robustness in high-dimensional constrained optimization problems.

### 3.4. Stochastic Binary Perturbation-Based Information Fusion Strategy

To mitigate premature convergence caused by excessive dependence on the global best solution, a stochastic binary perturbation-based information fusion strategy is proposed [33].

This mechanism combines dimension-wise selective perturbation with information fusion from non-elite individuals. The update rule is defined as:

(19) $$X_{\mathrm{new}}\left(i,:\right)=U_{1}\cdot X\left(i,:\right)+\left(1-U_{1}\right)\cdot \left(y+rand\cdot \left(X\left(r_{1},:\right)-X\left(r_{2},:\right)\right)\right)$$

where:

- $U_{1}=\left(rand(1,Dim)>r\right)$ is a binary vector controlling dimension-wise updates. It performs dimension-wise selective inheritance from the current individual, allowing part of the original solution structure to be preserved while perturbing only selected dimensions. This selective information fusion mechanism reduces destructive updates and improves search stability during local escape.
- $y=\left(X\left(i,:\right)+X\left(r_{3},:\right)\right)/2$.
- $r_{1},r_{2},r_{3}$ are randomly selected indices.

Compared with the original PKO, which tends to reinforce the region of the current global best and suffers from premature convergence, the proposed strategy introduces controlled stochasticity while preserving partial solution structure. By incorporating information from multiple randomly selected individuals and applying dimension-wise binary perturbations, it prevents search trajectory homogenization. This leads to enhanced ability to escape local optima and sustained population diversity throughout the optimization process.

### 3.5. Centroid-Based Adaptive Boundary Handling Mechanism

To ensure feasibility under boundary constraints, a centroid-based adaptive boundary repair mechanism is introduced. Unlike direct truncation, this method utilizes population distribution information to perform adaptive correction [34].

First, boundary violations are detected:

(20) $$flag_{ub}=X\left(i,:\right)>ub,\;flag_{lb}=X\left(i,:\right)<lb$$

The population centroid is computed as:

(21) $$pop_{mean}=mean\left(X,1\right)$$

For violated dimensions, repair is performed as:

(22) $$X\left(i,flag_{ub}\right)=\frac{pop_{mean}\left(flag_{ub}\right)+ub\left(flag_{ub}\right)}{2}$$

(23) $$X\left(i,flag_{lb}\right)=\frac{pop_{mean}\left(flag_{lb}\right)+lb\left(flag_{lb}\right)}{2}$$

Unlike conventional boundary clipping strategies that directly project infeasible solutions onto boundary limits, the proposed mechanism preserves partial population distribution characteristics through centroid-guided relocation, thereby reducing abrupt positional distortion and maintaining search continuity near constrained regions.

Compared with the original PKO, which applies direct boundary truncation without considering population distribution, the proposed centroid-based adaptive repair mechanism introduces a selective and distribution-aware feasibility control strategy. Instead of simple truncation, infeasible solutions are adaptively relocated toward the midpoint between the population centroid and the violated boundary, thereby preserving useful directional information while ensuring constraint satisfaction. This leads to enhanced robustness under bounded constraints, maintained population coherence, and prevention of disruptive boundary corrections, enabling stable optimization performance in constrained and real-world engineering applications.

### 3.6. Novelty and Methodological Contributions

It should be noted that some individual enhancement strategies adopted in IPKO, such as chaotic initialization, adaptive perturbation, and boundary repair mechanisms, have been previously investigated in swarm intelligence and metaheuristic optimization literature. Therefore, the primary contribution of this work does not lie in proposing entirely isolated new operators. Instead, the novelty of IPKO is reflected in the following methodological aspects.

#### 3.6.1. Phase-Deficiency-Oriented Integration

Unlike conventional hybrid metaheuristics that directly combine multiple independent strategies (e.g., chaotic mapping followed by opposition-based learning without stage-specific justification), IPKO is constructed based on a phase-deficiency-oriented redesign framework. Each enhancement mechanism is specifically developed according to a corresponding weakness identified in different search stages of the original PKO, including initialization, exploration, exploitation, and constraint handling. Consequently, the proposed framework establishes explicit correspondence between algorithmic deficiencies and adaptive search behaviors.

#### 3.6.2. Coordinated Adaptive Synergy

The proposed mechanisms are not introduced as isolated operators, but are cooperatively integrated into a coordinated adaptive search framework. The initialization strategy improves early-stage population diversity, the adaptive directional search mechanism strengthens guided exploration and exploitation transition, the stochastic binary perturbation strategy enhances local-optimum escape capability, and the centroid-based repair mechanism improves feasibility preservation under constrained boundaries. The interaction among these mechanisms forms a dynamically coordinated optimization process rather than a simple operator-level accumulation.

#### 3.6.3. Comparison with Recent Algorithms

To justify originality, we compare IPKO with several representative optimizers, including both classical metaheuristics and state-of-the-art adaptive algorithms. Table 2 summarizes the key differences in terms of strategy types, integration approach, stage-specific design, and adaptive coordination.

As shown in Table 2, classical algorithms (WOA, GWO, DE, PKO) rely on a single search mechanism without multi-strategy integration. Recent algorithms such as NRBO, KO, POA, HBA, and GBO introduce slightly improved mechanisms but still lack explicit stage-specific deficiency mapping. Even advanced adaptive algorithms like L-SHADE and CMA-ES, while employing sophisticated parameter adaptation, do not establish a one-to-one correspondence between each enhancement component and a specific weakness of the base optimizer. In contrast, IPKO explicitly links chaotic initialization to poor initial diversity, adaptive directional search to insufficient guidance during exploration, stochastic perturbation to local trapping, and centroid-based repair to boundary violation. Moreover, the activation conditions (predation efficiency PE and stagnation detection) coordinate the cooperation among strategies, which is absent in most existing approaches.

#### 3.6.4. Biologically Interpretable Behavior-Mechanism Coupling

From a biomimetic perspective, the proposed framework establishes behavior-mechanism coupling between pied kingfisher hunting behaviors and optimization dynamics. Specifically, diversified perch selection behavior motivates population initialization, hovering gaze adjustment behavior inspires directional neighborhood search, evasive movement after failed predation motivates adaptive perturbation, and territory relocation behavior motivates adaptive boundary handling. This biologically interpretable mechanism coordination distinguishes IPKO from conventional heuristic strategy stacking.

#### 3.6.5. Targeted Design for High-Dimensional Constrained Optimization

Finally, the proposed framework is specifically designed for high-dimensional constrained optimization problems. By integrating adaptive directional regulation, multi-source information fusion, and distribution-aware feasibility handling, IPKO improves convergence robustness, population diversity preservation, and constrained search adaptability in complex optimization landscapes.

Therefore, the contribution of this work lies not merely in combining existing techniques, but in constructing a biologically inspired, phase-deficiency-oriented, and adaptively coordinated optimization framework tailored to the specific weaknesses of PKO and the challenges of high-dimensional constrained optimization.

### 3.7. Summary of IPKO

As shown in Table 3, the proposed IPKO integrates four complementary mechanisms into a structured optimization pipeline. Each component explicitly targets a specific deficiency of the original PKO, collectively improving robustness, convergence behavior, and applicability to complex engineering optimization problems.

### 3.8. IPKO Algorithm Processes

The pseudocode workflow of the IPKO algorithm is presented in Algorithm 1.
**Algorithm 1** Improved Kingfisher Optimization (IPKO) Algorithm  1:Step 1: Sine chaotic opposition-based initialization (Equations (5)–(10))  2:Initialize population *X*, evaluate fitness, find $G_{\mathrm{best}}$, $f_{\mathrm{best}}$  3:Set stuck=0  4:Step 2: Main loop  5:**for** t=1 to MaxIter **do**  6:   **for** i=1 to *N* **do**  7:     Step 3: Adaptive directional search  8:     Compute PE  9:     **if** rand() ≤(1−PE)(Equation(11)) **then**10:        $r_{1}=\mathrm{randi}\left(\left[1,N\right]\right)$, r=rand()11:        $X_{2}=\left(G_{\mathrm{best}}-X_{i}^{\left(t-1\right)}\right)\times r$12:        $B=2\times \cos(\frac{\pi }{2}\times \left(1-\frac{t}{MaxIter}\right))$13:        θ=⌈360×rand()⌉14:        V=randi([2,5])15:        $X_{\mathrm{new}}=X_{2}+\left(X_{r_{1}}^{\left(t-1\right)}-X_{i}^{\left(t-1\right)}\right)\times \cos\theta \times B\times V\times rand\left(\right)$16:        $\;\;+X_{r_{1}}^{\left(t-1\right)}\times \sin\theta \times B\times rand\left(\right)$17:        $X_{i}^{\left(t\right)}=\mathrm{CentroidBoundary}\left(X_{\mathrm{new}}\right)$ (Equations (20)–(23))18:     **else**19:        $X_{i}^{\left(t\right)}=\mathrm{OriginalPKODiving}\left(X_{i}^{\left(t-1\right)}\right)$20:        $X_{i}^{\left(t\right)}=\mathrm{CentroidBoundary}\left(X_{i}^{\left(t\right)}\right)$21:     **end if**22:     Step 4: Stochastic binary perturbation fusion23:     **if** $f\left(X_{i}^{\left(t\right)}\right)<f_{\mathrm{best}}$ **then**24:        stuck=0, $G_{\mathrm{best}}=X_{i}^{\left(t\right)}$, $f_{\mathrm{best}}=f\left(X_{i}^{\left(t\right)}\right)$25:     **else**26:        stuck=stuck+127:     **end if**28:     **if** stuck≥5 **then**29:        Choose $r_{1},r_{2},r_{3}$ distinct and ≠i30:        $y=(X_{i}^{\left(t\right)}+X_{r_{3}}^{\left(t\right)})/2$31:        $U_{1}=\left(rand\left(1,Dim\right)>0.5\right)$32:        $X_{\mathrm{new}}=U_{1}\cdot X_{i}^{\left(t\right)}+\left(1-U_{1}\right)\cdot \left(y+rand\left(\right)\cdot \left(X_{r_{1}}^{\left(t\right)}-X_{r_{2}}^{\left(t\right)}\right)\right)$ (Equation (19))33:        $X_{i}^{\left(t\right)}=\mathrm{CentroidBoundary}\left(X_{\mathrm{new}}\right)$34:        stuck=035:     **end if**36:   **end for**37:**end for**38:return $G_{\mathrm{best}}$, $f_{\mathrm{best}}$

### 3.9. Complexity Analysis of the IPKO Algorithm

Algorithmic complexity is a critical indicator of computational feasibility and scalability, commonly evaluated in terms of time complexity (runtime cost) and space complexity (memory usage) [37]. An effective algorithmic enhancement should improve convergence behavior and solution quality without introducing additional asymptotic computational burden beyond that of the baseline method [38].

#### 3.9.1. Time Complexity Analysis

The time complexity of IPKO is analyzed by decomposing the algorithm into the initialization phase and the iterative optimization phase.

##### Initialization Phase

The initialization phase employs sinusoidal chaotic mapping combined with opposition-based learning to generate the initial population. For a population size of *N* in a *D*-dimensional search space, chaotic sequence generation, opposition solution construction, and fitness evaluation all require linear operations with respect to both *N* and *D*. Therefore, the total computational cost of the initialization phase is: $O_{\mathrm{init}}=O\left(N\cdot D\right)$.

##### Main Iterative Phase

The main optimization loop is executed for $T_{\max}$ iterations. In each iteration, the following operations are performed:

- Position updating through exploration or exploitation strategies;
- Adaptive Directional Search Mechanism (conditionally triggered);
- Stochastic binary perturbation-based information fusion;
- Centroid-based adaptive boundary handling;
- Fitness evaluation of updated individuals.

All position update, perturbation, and repair operations are applied on a dimension-wise basis and involve simple arithmetic computations. Even though some mechanisms (e.g., environmental update and stochastic perturbation) are conditionally activated, their worst-case computational cost per iteration remains linear with respect to population size and dimensionality. Consequently, the per-iteration complexity is bounded by: $O_{\mathrm{iter}}=O\left(N\cdot D\right)$. Thus, the total time complexity of the iterative phase is: $O_{\mathrm{loop}}=O\left(T_{\max}\cdot N\cdot D\right)$.

##### Overall Time Complexity

By combining the initialization and iterative phases, the overall time complexity of IPKO is given by:

(24) $$O_{\mathrm{total}}=O\left(N\cdot D\right)+O\left(T_{\max}\cdot N\cdot D\right)=O\left(T_{\max}\cdot N\cdot D\right).$$

#### 3.9.2. Space Complexity Analysis

The space complexity of IPKO is primarily determined by the storage requirements of population-related variables.

##### Initialization Phase

During initialization, the algorithm stores the population matrix and corresponding fitness values, requiring: $O_{\mathrm{init}}=O\left(N\cdot D\right)$.

##### Main Iterative Phase

During the optimization process, IPKO maintains the current population, temporary candidate solutions, and the population centroid vector. The centroid requires O(D) memory, which is negligible compared to the dominant population storage. Hence, the total memory requirement remains: $O_{\mathrm{loop}}=O\left(N\cdot D\right)$.

##### Overall Space Complexity

Combining both phases, the overall space complexity of IPKO is:

(25) $$O_{\mathrm{total}}=O\left(N\cdot D\right).$$

#### 3.9.3. Comparative Complexity Discussion

Compared with the original PKO algorithm, IPKO exhibits identical asymptotic time and space complexities, namely $O(T_{\max}\cdot N\cdot D)$ for time and O(N·D) for space. Although IPKO introduces multiple enhancement strategies, including chaotic initialization, adaptive directional search, diversity-preserving perturbation, and centroid-based handling, these components only incur constant-factor computational overhead and do not alter the algorithm’s asymptotic complexity. Therefore, IPKO achieves improved convergence quality and robustness while maintaining the lightweight computational characteristics and scalability of the baseline PKO.

## 4. Results on Benchmark Functions

### 4.1. Experimental Setup

#### 4.1.1. Benchmark Functions and Comparative Algorithms

To comprehensively evaluate the effectiveness and robustness of the IPKO algorithm in comparison to the original PKO algorithm, this study conducted comparative experiments using all 29 benchmark functions from the CEC2017 test suite across 10, 30, 50, and 100 dimensions. These functions are systematically classified into four distinct groups: (1) Unimodal Functions (F1–F10): These fundamental functions are mainly intended for evaluating the algorithm’s local search capacity and convergence rate. (2) High-Condition-Number Functions (F11–F20): Characterized by notably disparate parameter scales, this category examines the algorithm’s proficiency in dealing with imbalanced and ill-conditioned search landscapes. (3) Composite Multimodal Functions (F21–F25): These functions amalgamate multiple basic functions to mimic intricate, multi-faceted search scenarios and pose a challenge to the algorithm’s ability to evade local optima. (4) Hybrid Composition Functions (F26–F29): By integrating diverse function characteristics, this category offers a rigorous assessment for evaluating the overall global optimization performance and robustness.

The performance of the proposed IPKO algorithm was comprehensively compared with that of 11 well-established optimization algorithms, namely the original (PKO), Newton-Raphson Based Optimizer (NRBO) [39], K-means Optimizer (KO) [40], Pelican Optimization Algorithm (POA) [41], Honey Badger Algorithm (HBA) [42], Gradient-Based Optimizer (GBO) [43], Whale Optimization Algorithm (WOA), Grey Wolf Optimizer (GWO), Linear Population Size Reduction SHADE (L-SHADE), Covariance Matrix Adaptation Evolution Strategy (CMA-ES), and Differential Evolution (DE). Although some of these algorithms are newly developed, they have been thoroughly validated in their corresponding sources and exhibit strong competitive capabilities, thus serving as appropriate and challenging benchmarks for comparison.

#### 4.1.2. Parameter Settings and Experimental Environment

To ensure a fair, reproducible, and statistically reliable evaluation, all comparative experiments were conducted under identical experimental settings. For all algorithms, the population size was fixed at N=30, and the maximum number of iterations was set to $T_{\max}=500$, resulting in an identical upper bound on the total number of function evaluations across methods. Each algorithm was independently executed 30 times on every benchmark function to alleviate the influence of stochastic variability. From these runs, mean value, standard deviation, and average runtime (art) were recorded as performance indicators, providing a comprehensive assessment in terms of accuracy, robustness, and computational efficiency. To guarantee fairness in comparison, all competing algorithms employed their original parameter settings as recommended in the corresponding literature. No problem-specific parameter tuning was performed for any algorithm, ensuring that performance differences arise from algorithmic mechanisms rather than manual optimization. All simulations were implemented in MATLAB R2024a and executed on a 64-bit Windows 11 operating system. The hardware platform consisted of a 13th Gen Intel^®^ Core i7–13700H CPU operating at 2.40 GHz with 16 GB of RAM. To enhance reproducibility, all algorithms were executed under the same software environment and computational resources.

All experiments were repeated 30 independent runs per problem instance. Since the performance data of stochastic metaheuristics typically violate normality assumptions, we employ non-parametric statistical tests following standard practice in evolutionary computation. Specifically, the Friedman test is used to detect overall significant differences among multiple algorithms across benchmark functions. When the Friedman test rejects the null hypothesis (p<0.05), we apply Holm’s post-hoc procedure to control the family-wise error rate in pairwise comparisons between IPKO and each competitor. All reported *p*-values are two-tailed with a significance level α=0.05.

### 4.2. Comprehensive Performance Evaluation on CEC2017

The comprehensive comparative results of all twelve algorithms across the 29 test functions are presented in the subsequent sections.

#### 4.2.1. Numerical Results and Statistical Analysis

The comparative performance of IPKO against 11 state-of-the-art algorithms on the CEC2017 benchmark suite is evaluated across four dimensional settings: 10D, 30D, 50D, and 100D. The numerical results for each dimension are presented in Table 4, Table 5, Table 6 and Table 7, where Table 4 reports 30D results, Table 5 reports 10D results, Table 6 reports 50D results, and Table 7 report 100D results. To statistically validate the overall performance differences, the Friedman test is conducted for each dimension, with results summarized in Table 8, Table 9, Table 10 and Table 11. The corresponding Holm post-hoc test results for each dimension are presented in Table 12, Table 13, Table 14 and Table 15, where the proposed IPKO is compared with other competitor algorithms.

- Overall Performance Analysis

The numerical results demonstrate that IPKO consistently achieves superior optimization performance across the majority of benchmark functions. In terms of solution accuracy, IPKO obtains the best mean values on 14, 17, 17, and 15 functions in the 10D, 30D, 50D, and 100D cases, respectively. These results indicate that IPKO is capable of producing high-quality solutions across diverse optimization landscapes. Regarding solution stability, IPKO also demonstrates competitive robustness. It achieves the lowest standard deviation on 8 functions (10D), 10 functions (30D), 4 functions (50D), and 3 functions (100D). These consistently low variance values indicate that the algorithm maintains stable convergence behavior and reliable performance across independent runs.

Notably, the superiority of IPKO becomes particularly evident on multimodal and hybrid benchmark functions, which typically exhibit complex and challenging search landscapes. This improvement can be attributed to the proposed enhancement strategies, including sine chaotic initialization and stochastic binary perturbation, which increase population diversity and enable the algorithm to escape local optima more effectively.

B. Scalability with Increasing Dimensionality

Another important observation is that the performance advantage of IPKO remains stable as the problem dimensionality increases. Although optimization difficulty increases significantly when the dimension grows from 10D to 100D, IPKO still maintains a high number of best-performing functions in terms of both mean and standard deviation.

For example, even in the 100D scenario, which represents the most challenging case, IPKO still achieves the best mean results on 15 benchmark functions. This demonstrates that the proposed algorithm maintains strong scalability and robustness when dealing with high-dimensional optimization problems. This sustained performance advantage indicates that the proposed mechanisms—particularly adaptive directional search and centroid-based handling—help maintain population diversity and guide individuals toward promising regions while avoiding premature convergence.

C. Friedman Statistical Test and Discussion

To further verify the statistical significance of the observed performance differences, the Friedman test is conducted across all benchmark functions. The results show highly significant differences among the compared algorithms (p≪0.001). IPKO consistently achieves the lowest average ranks of 2.25, 1.93, 1.64, and 2 for the 10D, 30D, 50D, and 100D cases, respectively, indicating the best overall performance.

The ranking results reveal that LSHADE generally maintains competitive positions, while GBO shows improved performance as dimensionality increases. In contrast, the baseline PKO exhibits relatively weaker rankings in higher-dimensional scenarios, highlighting the effectiveness of the proposed enhancement strategies.

Further Holm post-hoc tests (α=0.05) show that IPKO significantly outperforms all or the vast majority of the compared algorithms (all adjusted *p*-values are less than 0.05, with only no significant difference from PKO and LSHADE in a few dimensions). This fully confirms that the proposed improved strategies enable IPKO to achieve a significant and stable advantage in overall optimization performance.

Overall, the consistently lowest average ranks and highly significant *p*-values confirm that the superior performance of IPKO is statistically reliable rather than incidental, demonstrating its strong optimization capability and robustness on the challenging CEC2017 benchmark suite.

#### 4.2.2. Box Plot Behavior Analysis (D = 30)

**Figure 3:**
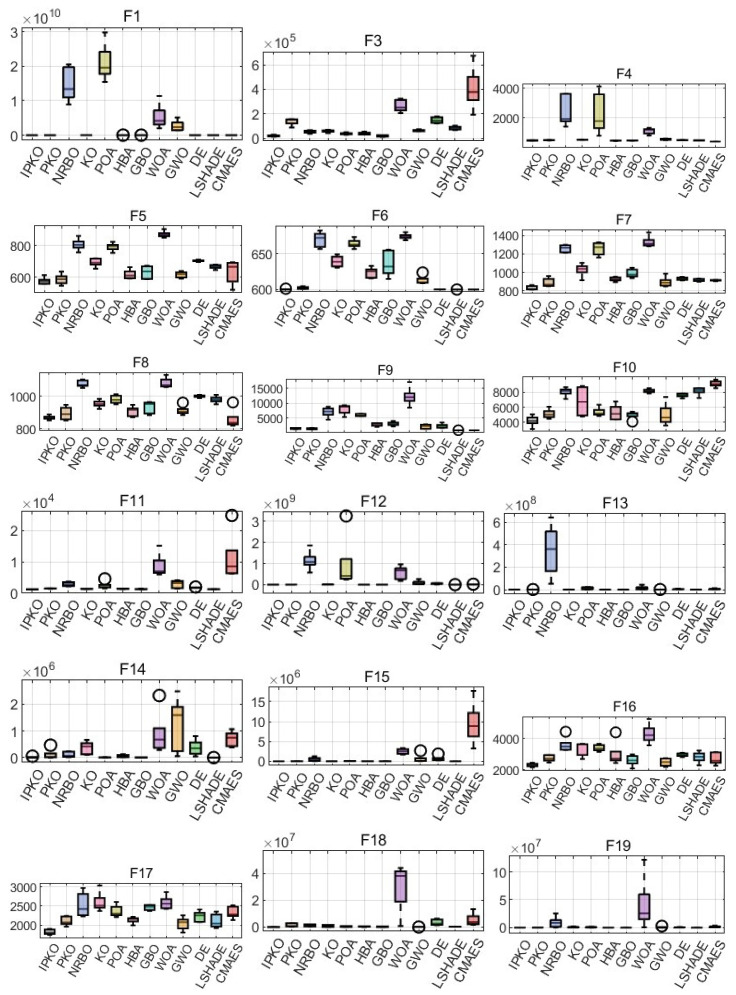
Box plots of 12 algorithms on F1, F3– F19 test functions (D = 30).

**Figure 4:**
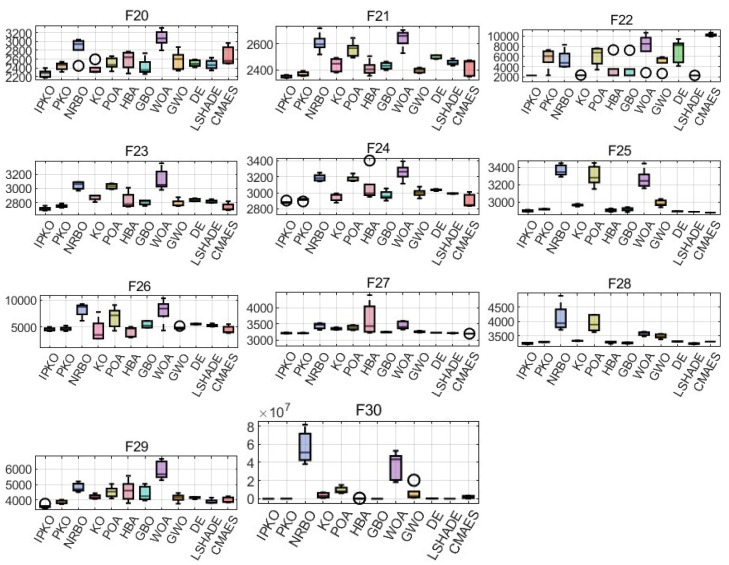
Box plots of 12 algorithms on F20-F30 test functions (D = 30).

As shown in the box plot analysis (Figure 3 and Figure 4), IPKO generally exhibits competitive performance in terms of both accuracy and robustness compared with the other algorithms. For unimodal functions (e.g., F1 and F3), IPKO achieves relatively low fitness values with a compact distribution, indicating stable convergence behavior. While some algorithms such as NRBO or DE occasionally reach comparable results, several methods (e.g., WOA and GWO) show larger dispersion or higher median values, suggesting less reliable convergence. In multimodal scenarios (e.g., F7, F12, F14, and F19), IPKO maintains a good balance between exploration and exploitation. It consistently ranks among the top-performing algorithms and demonstrates relatively stable distributions. In contrast, certain algorithms such as POA and HBA exhibit wider interquartile ranges or outliers, indicating susceptibility to local optima. For more complex or high-dimensional functions (e.g., F25 and F30), IPKO continues to show strong robustness, with comparatively low variance and competitive median performance. Although some algorithms (e.g., CMA-ES or LSHADE variants) achieve similar or occasionally better results on specific functions, IPKO maintains more consistent behavior across different problem types. Overall, the box plots indicate that IPKO provides a favorable trade-off between solution quality and stability, particularly in complex and multimodal optimization landscapes.

#### 4.2.3. Convergence Analysis (D = 30)

**Figure 5:**
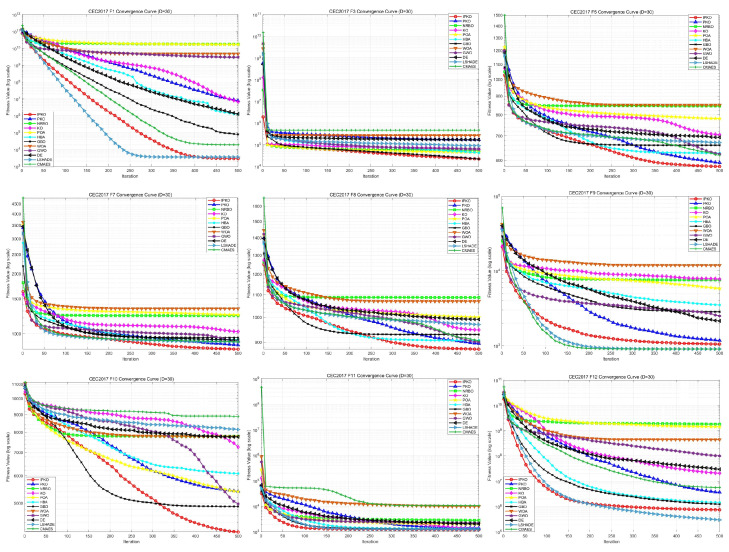

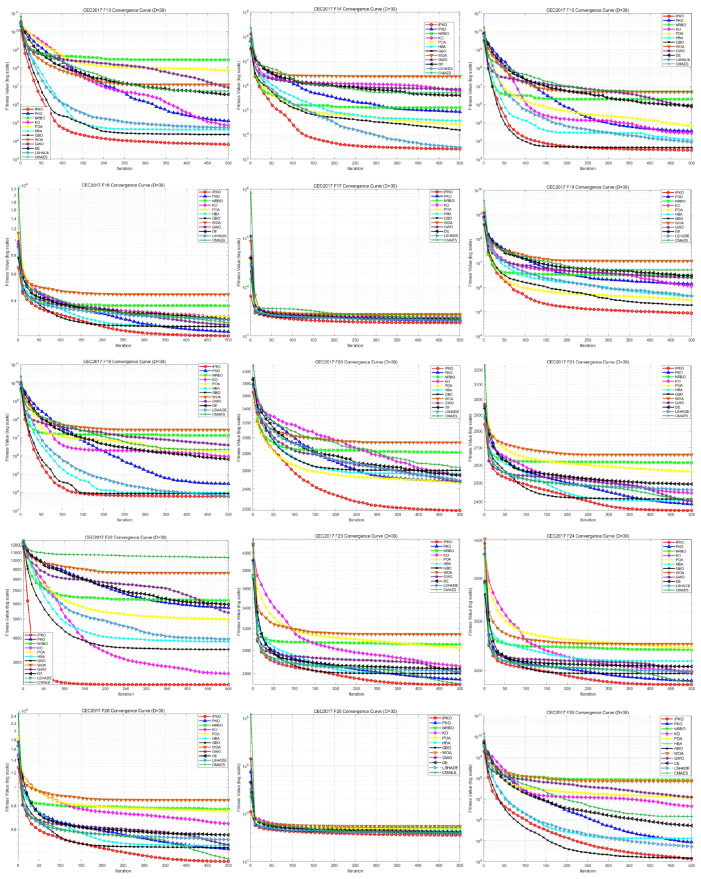
Convergence curves of 12 algorithms on cec2017 test functions (D = 30).

The convergence behaviors of all algorithms on the CEC2017 benchmark suite are illustrated in Figure 5. Overall, IPKO demonstrates stable and competitive convergence behavior across diverse function types.

On unimodal functions (e.g., F1 and F5), IPKO exhibits faster early-stage convergence than most competing algorithms, indicating that its initialization and adaptive mechanisms effectively guide the population toward promising search regions. For multimodal functions (e.g., F9 and F10), IPKO maintains optimization momentum in later stages, continuing to refine solutions while several baseline methods experience convergence stagnation.

The advantage becomes more evident on hybrid functions (e.g., F16–F24), where IPKO consistently reduces fitness values throughout the search process, while comparative algorithms tend to reach convergence plateaus at earlier stages. On highly challenging functions (e.g., F22 and F30), IPKO achieves sustained fitness reduction despite premature convergence observed in several baseline algorithms.

Overall, IPKO exhibits strong convergence characteristics in both early-stage search efficiency and late-stage solution refinement. These observations further support the effectiveness of the proposed mechanisms in enhancing both the exploration capability and exploitation efficiency of the algorithm.

#### 4.2.4. Computational Efficiency and Robustness Analysis

Beyond solution accuracy, computational efficiency is also an important criterion for evaluating metaheuristic algorithms. This section investigates the efficiency of IPKO from two perspectives: actual runtime and performance under limited function evaluation (FEs) budgets.

- Runtime Analysis

The empirical runtime analysis was conducted under the same experimental settings described previously. Table 4 report the average runtime (art) of each algorithm on the 30 CEC2017 benchmark functions, while the overall mean runtime and standard deviation are summarized in Table 16.

As shown in Table 16, IPKO requires more runtime than the original PKO due to the additional mechanisms introduced in the proposed framework, including chaotic initialization, adaptive directional search, stochastic perturbation, and centroid-based boundary handling. The mean runtime of IPKO is approximately 2.15 times that of PKO (0.768 s versus 0.358 s). However, these operations mainly consist of population-level vector computations and therefore only introduce constant-factor overhead. Consequently, the asymptotic computational complexity remains unchanged:

(26) $$O(T_{\max}\cdot N\cdot D)$$

where $T_{\max}$ denotes the maximum number of iterations, *N* is the population size, and *D* is the problem dimension.

**Figure 6:**
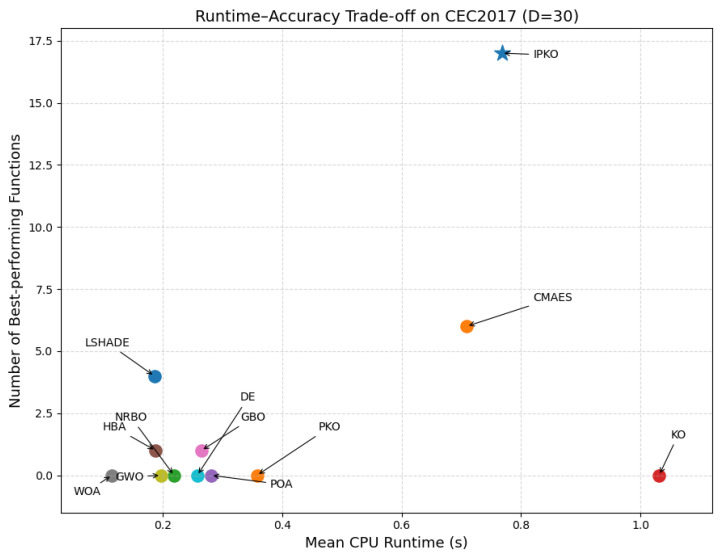
Runtime–accuracy trade-off of the compared algorithms on CEC2017 benchmark functions (D = 30). The vertical axis denotes the number of benchmark functions on which each algorithm achieved the best mean objective value.

To further analyze the trade-off between runtime and optimization performance, Figure 6 presents a runtime–accuracy scatter plot, where the horizontal axis represents the average CPU runtime and the vertical axis denotes the number of benchmark functions on which each algorithm achieves the best mean objective value. WOA and LSHADE exhibit relatively low runtime costs; however, their optimization performance remains inferior to that of IPKO on most benchmark functions. Although IPKO incurs a moderate runtime increase compared with lightweight algorithms, it achieves the largest number of best-performing results, indicating a favorable balance between computational cost and solution quality.

Compared with CMAES and KO, which also exhibit relatively high computational costs, IPKO provides superior optimization performance while maintaining a comparable runtime level. In particular, KO shows the highest average runtime (1.031 s) without achieving competitive solution quality, whereas IPKO obtains substantially better optimization results with lower computational cost. These observations indicate that the additional computational overhead introduced by IPKO is effectively translated into improved search capability and robustness.

Overall, the runtime analysis confirms that IPKO maintains the same asymptotic complexity as other population-based metaheuristics, while the measured runtime increase remains moderate relative to the achieved improvement in optimization performance and stability.

B. Performance Under Limited Function Evaluation Budgets

To further evaluate computational efficiency under constrained resources, experiments were conducted on the CEC2017 benchmark suite using FE budgets of {5000,10,000,15,000} under 30D, 50D, and 100D settings. Table 17 reports the corresponding average rankings.

The results show that IPKO achieves the best average rank in all settings (1.6–2.5), demonstrating stable performance across different dimensions and evaluation budgets. GBO and LSHADE generally occupy the second tier, whereas NRBO and WOA consistently rank among the weakest algorithms.

To quantify how effectively additional evaluations improve optimization performance, a Budget Efficiency Ratio (BER) is defined as

(27) $$\mathrm{BER}=\frac{f_{5000}-f_{15,000}}{f_{5000}}\times 100\%,$$

where $f_{5000}$ and $f_{15,000}$ denote the average fitness values obtained under FE budgets of 5000 and 15,000, respectively.

Table 18 reports the geometric mean of final errors and the corresponding BER values for four representative algorithms in 30D and 100D.

Among all compared algorithms, IPKO consistently achieves the lowest average errors under both small and large FE budgets. Notably, even at 5k evaluations, the error obtained by IPKO $\left(30D:6.42\times 10^{2};\;100D:2.34\times 10^{4}\right)$ remains lower than the final errors achieved by most competing algorithms at 15k evaluations, highlighting its strong early-stage search capability.

In terms of budget utilization efficiency, IPKO achieves BER values of approximately 66% in 30D and 85% in 100D, indicating that additional evaluations can be effectively exploited to further refine solution quality. The larger BER observed in 100D suggests that IPKO maintains strong scalability in high-dimensional search spaces.

GBO and LSHADE also achieve relatively high BER values in 30D (greater than 90%), indicating substantial improvements when additional evaluations are available. However, their final optimization errors at 15k evaluations remain approximately one order of magnitude higher than those of IPKO. In 100D, the BER values of GBO and LSHADE decrease to around 80%, implying reduced search efficiency under higher-dimensional optimization landscapes.

By contrast, WOA exhibits the weakest budget utilization capability, with BER values below 30% in both dimensions. Moreover, its optimization errors remain consistently two to three orders of magnitude larger than those of IPKO across all FE budgets, indicating limited suitability for optimization scenarios with strict evaluation constraints.

Overall, both the ranking analysis and BER evaluation consistently verify the effectiveness of IPKO under limited FE budgets. IPKO not only achieves superior solution quality during the early search stage, but also maintains stable convergence as additional evaluations become available. Although GBO and LSHADE become more competitive under larger budgets, they still fail to surpass IPKO in overall optimization accuracy.

C. Summary

The strong efficiency of IPKO under limited computational budgets can be attributed to the complementary roles of its enhancement mechanisms. Chaotic initialization improves early population diversity, adaptive guidance strengthens exploitation toward promising regions, and centroid-based boundary handling reduces ineffective evaluations caused by infeasible solutions. As a result, IPKO achieves a balanced combination of computational efficiency, budget utilization capability, and robust optimization performance.

#### 4.2.5. Parameter Sensitivity Analysis

To systematically evaluate the influence of key parameters on the performance of the proposed IPKO algorithm and to justify the parameter configuration adopted in the comparative experiments, a comprehensive sensitivity analysis is conducted on the population size (Pop) and the balance factor (*c*). The experiments are performed on eight representative functions from the CEC2017 benchmark suite (F1, F3, F7, F10, F13, F20, F23, F27) with a dimensionality of 30, covering unimodal, multimodal, hybrid, and composition optimization problems. All results are averaged over 30 independent runs.

- One-Dimensional Sensitivity Analysis

(1) Population Size

To examine the impact of population size, *c* is fixed at 2.0, while Pop varies from 20 to 80 with an increment of 5. The Friedman rankings across all test functions are summarized in Table 19.

The Friedman test yields a *p*-value of $1.68\times 10^{-4}$, which is significantly lower than 0.05, indicating that the differences among population sizes are statistically significant. As shown in Table 19, Pop = 25 achieves the best average rank (3.00), followed by Pop = 30 (3.75), demonstrating competitive performance.

Although Pop = 25 exhibits the best overall ranking, Pop = 30 provides a favorable trade-off between solution quality and computational cost. Therefore, Pop = 30 is selected as the default setting in subsequent experiments to ensure both efficiency and consistency with commonly adopted configurations in the literature.

(2) Balance Factor *c*

To evaluate the sensitivity to the balance factor, Pop is fixed at 30, while *c* varies from 0.5 to 3.0 with a step size of 0.5. The corresponding Friedman rankings are presented in Table 20.

The Friedman test produces a *p*-value of 0.623 (>0.05), indicating that no statistically significant differences are observed among the tested values of *c*. This suggests that the proposed IPKO algorithm is relatively insensitive to the choice of this parameter.

Among all candidates, *c* = 2.5 achieves the best average rank (2.50), followed by *c* = 2.0 (3.25). Considering both performance consistency and stability across different problem types, *c* = 2.0 is selected as the default setting. This observation further confirms the robustness of IPKO with respect to variations in the balance factor.

B. Two-Dimensional Sensitivity Analysis (Pop × c)

To further investigate the combined effect of the two parameters, a full grid search is conducted over Pop∈[20,80] and c∈[0.5,3.0]. The mean fitness values averaged over all test functions are reported in Table 21.

**Figure 7:**
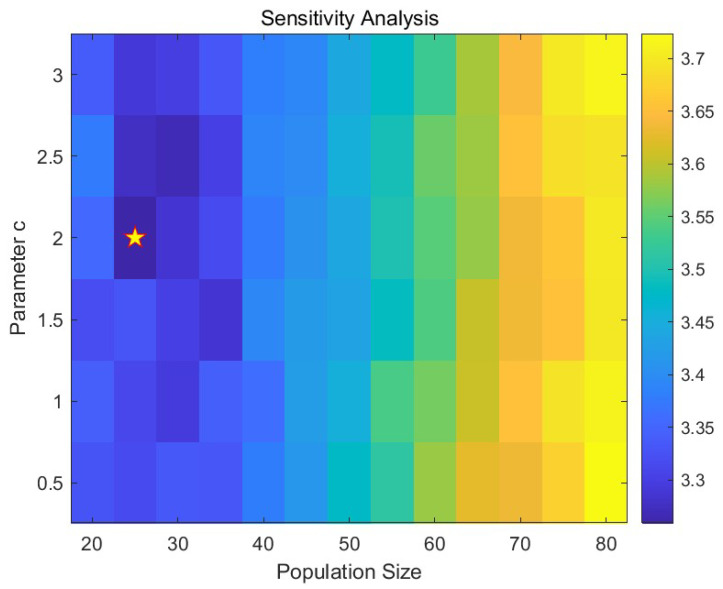
Heatmap of mean fitness values for different combinations of population size and balance factor.

In addition, the sensitivity landscape is visualized in Figure 7 using a heatmap representation, which provides an intuitive illustration of the performance distribution over the parameter space. The star marker highlights the globally optimal parameter combination.

From both Table 21 and Figure 7, several important observations can be made:

- The best performance is achieved at (Pop,*c*) = (25,2.0), yielding the lowest mean fitness value of 3.2587.
- A clear performance gradient can be observed along the population size axis, where larger populations generally lead to improved optimization results.
- In contrast, variations along the c−axis are relatively mild, indicating that the algorithm is less sensitive to this parameter.

These results suggest that the two parameters jointly influence the optimization performance, while the population size plays a more dominant role.

Nevertheless, the default configuration adopted in this study, (Pop,*c*) = (30,2.0), achieves a mean fitness value of 3.2862, resulting in a marginal performance gap of only 0.84% compared to the global optimum. This negligible difference demonstrates that the selected parameter setting is near-optimal.

C. Summary of Parameter Settings

Based on the above analyses, the parameter configuration used in the subsequent experiments is determined as follows:

- Pop = 30: This setting achieves competitive performance in the one-dimensional analysis while ensuring a fair and unbiased comparison with existing algorithms.
- *c* = 2.0: This value provides stable and consistent performance across all test functions and exhibits strong robustness.

The two-dimensional analysis further confirms that this configuration achieves performance within 0.84% of the global optimum, validating its effectiveness as a well-balanced and reliable default setting.

### 4.3. Behavioral and Mechanism Analysis

#### 4.3.1. Ablation Study (D = 30)

To further examine the contribution of each enhancement strategy, a progressive ablation study was conducted. The experiment settings are the same as the previous experiments. Specifically, four algorithmic variants were constructed: IPKOa (only sine chaotic opposition-based learning), IPKOab (with the addition of the Adaptive Directional Search Mechanism), IPKOabc (further incorporating the stochastic binary perturbation-based information fusion), and the complete IPKO, which additionally includes the centroid-based adaptive boundary handling mechanism. The baseline PKO was also included for comparison. All variants were run 30 independent times on ten representative CEC2017 functions (F3, F4, F5, F8, F9, F10, F14, F18, F24, F28) under 30 dimensions.

- Quantitative Results

Table 22 presents the mean, standard deviation, and within-function ranking results (lower rank indicates better performance) for all algorithmic variants. In addition, the Wilcoxon signed-rank test at a significance level of 0.05 was employed to evaluate the statistical significance between each variant and the complete IPKO.

Several important observations can be drawn from the results:

- IPKO consistently achieves the best mean fitness values on all benchmark functions, obtaining an average rank of 1.00. This result demonstrates the superior optimization capability and robustness of the proposed complete framework.
- IPKOabc ranks second overall with an average rank of 2.00. This variant excludes only the centroid-based adaptive boundary handling mechanism. Moreover, no statistically significant difference is observed between IPKOabc and IPKO on five functions (F3, F14, F18, F24, and F28), indicating that the first three enhancement strategies already provide substantial optimization improvements. However, the remaining performance gap on the other functions further confirms the importance of the proposed boundary handling mechanism in maintaining search stability and solution quality.
- PKO, IPKOa, and IPKOab are all significantly inferior to IPKO on every benchmark function (p<0.05). Their average ranks range from 3.70 to 4.20, which are considerably worse than those of IPKO and IPKOabc. This finding demonstrates that the four proposed strategies complement each other effectively and should be integrated synergistically to achieve optimal performance.

B. Convergence Behavior

**Figure 8:**
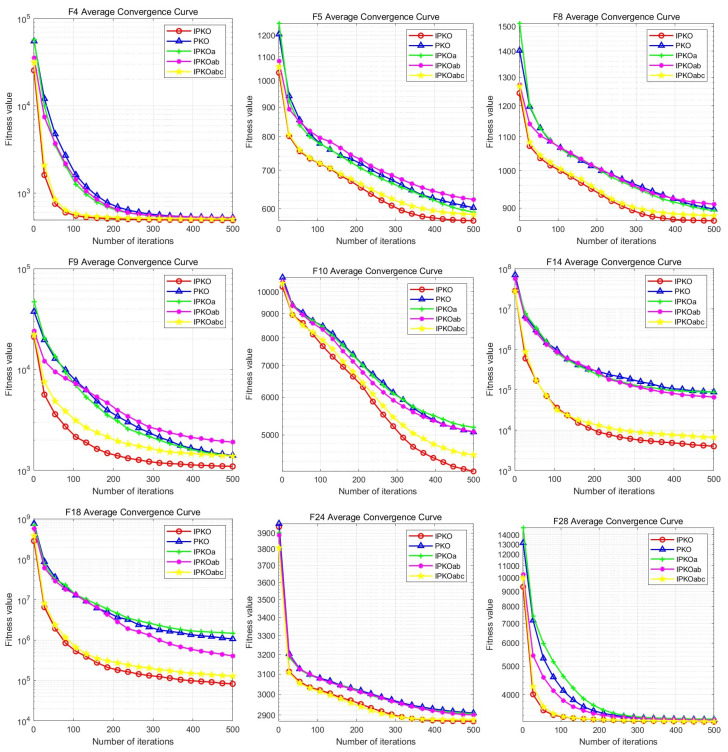
Ablation study (D = 30).

The convergence behaviors of all algorithmic variants are illustrated in Figure 8. It can be observed that IPKOa achieves noticeably faster convergence during the early search stage compared with the original PKO, indicating that the sine chaotic opposition-based learning strategy effectively enhances population diversity and exploration ability.

After introducing the Adaptive Directional Search Mechanism, IPKOab exhibits improved convergence speed during the middle stage of the optimization process, reflecting stronger local exploitation capability and improved search guidance.

Furthermore, the incorporation of the stochastic binary perturbation-based information fusion strategy in IPKOabc effectively alleviates premature convergence and enables continuous fitness improvement during the later iterations.

Among all variants, the complete IPKO consistently demonstrates the fastest convergence rate and achieves the lowest final fitness values. This superior performance can be attributed to the centroid-based adaptive boundary handling mechanism, which improves population feasibility, stabilizes the search dynamics, and enhances the balance between exploration and exploitation.

Overall, the progressive performance improvements observed from IPKOa to the complete IPKO clearly demonstrate that each proposed enhancement strategy contributes positively to the optimization process. The quantitative results and convergence analyses jointly verify the effectiveness and rationality of the integrated IPKO framework.

#### 4.3.2. Search Behavior Analysis

Population diversity and the balance between exploration and exploitation are two critical factors influencing the effectiveness of metaheuristic optimization algorithms. In the proposed IPKO, both aspects are enhanced through several complementary mechanisms that shape the search dynamics throughout the optimization process.

In terms of population diversity, IPKO implicitly maintains diverse search patterns through multiple strategies. During the initialization stage, the sine chaotic opposition-based initialization generates a well-dispersed population, enabling broader coverage of the search space. During the optimization process, the Adaptive Directional Search Mechanism and stochastic binary perturbation introduce dimension-wise and directional variations, continuously injecting new search directions and preventing rapid diversity loss commonly observed in swarm-based algorithms. In addition, the centroid-based handling mechanism preserves population coherence without forcing individuals toward rigid boundaries, thereby avoiding abrupt diversity collapse.

Based on the maintained diversity, IPKO further achieves an effective balance between exploration and exploitation across different search stages. In the early stage, exploration is emphasized through chaotic initialization and large-amplitude stochastic updates, enabling rapid traversal of the global search space. As the optimization progresses, the information fusion strategy guides individuals toward high-quality regions while preserving selective randomness at the dimension level, allowing effective solution refinement.

Overall, this dynamic search behavior enables IPKO to maintain fast early-stage convergence while preserving refinement capability in later iterations, which explains its strong performance on complex multimodal and high-dimensional optimization problems.

#### 4.3.3. Relationship Between Improvement Strategies and Experimental Performance

**Figure 9:**
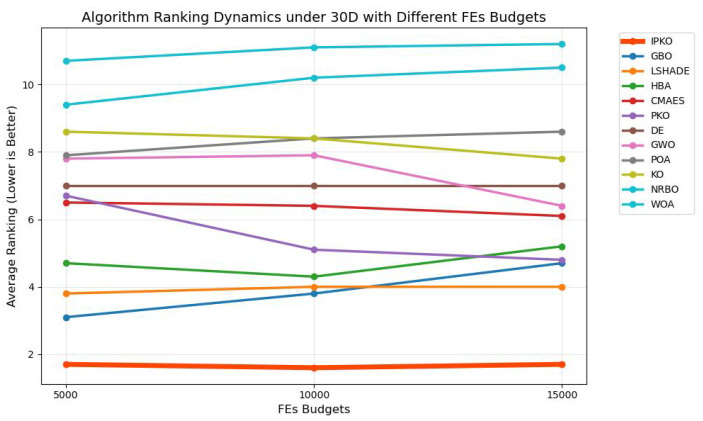
Algorithm ranking dynamics under 30D.

**Figure 10:**
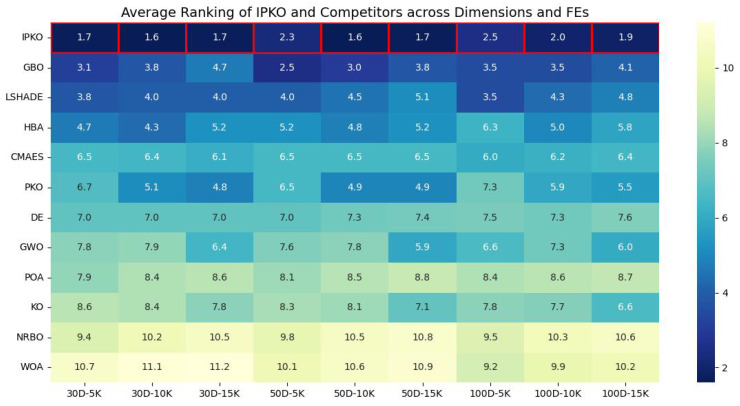
Dimension comparison heatmap.

**Figure 11:**
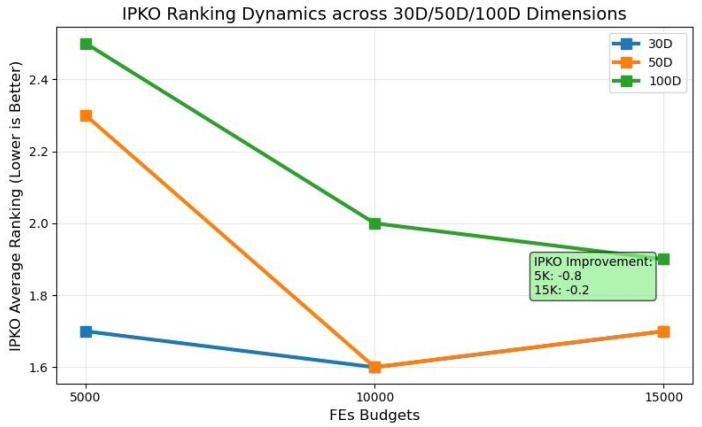
IPKO ranking dynamics across dimensions.

To visually demonstrate how the proposed enhancement strategies contribute to IPKO’s experimental performance, Figure 9, Figure 10 and Figure 11 illustrate the ranking dynamics of all algorithms under varying computational budgets and dimensional settings.

- Competitive Advantage Under Limited Budgets

Figure 9 shows the average ranking dynamics at 30D under increasing FEs budgets. IPKO maintains the lowest rank across all budget levels (1.6→1.7), demonstrating strong early exploration enabled by chaotic initialization and continued refinement from its adaptive mechanisms. In contrast, WOA and NRBO exhibit higher ranks with minimal budget sensitivity, indicating ineffective use of additional evaluations.

B. Comprehensive Performance Landscape

The heatmap in Figure 10 presents average rankings across three dimensions and three budgets. IPKO consistently occupies the darkest blue region (lowest ranks) across all twelve configurations, confirming robust performance regardless of dimensionality. LSHADE and GBO emerge as the closest competitors, while PKO consistently lags behind IPKO—directly quantifying the benefit of the proposed enhancements.

C. Dimensional Scalability of IPKO

Figure 11 shows that IPKO’s average rank slightly increases with dimensionality at low FEs (e.g., from 1.7 in 30D to 2.5 in 100D at 5K), reflecting increased optimization difficulty. However, with increasing FEs, the ranking improves consistently and the performance gap across dimensions narrows, demonstrating strong scalability and effective utilization of computational resources.

D. Summary

Figure 9, Figure 10 and Figure 11 establish a clear link between IPKO’s enhancement strategies and its experimental performance: chaotic initialization enables strong early exploration, adaptive mechanisms maintain search effectiveness across budgets, and continued refinement ensures improvement with increasing evaluations—particularly in high-dimensional spaces.

### 4.4. Experimental Summary

The experimental results on the CEC2017 benchmark suite demonstrate that the proposed IPKO algorithm consistently outperforms eleven state-of-the-art metaheuristic algorithms in terms of solution accuracy, convergence speed, and stability, particularly on high-dimensional and multimodal problems.

These performance gains arise from the synergistic interaction of multiple enhancement mechanisms rather than any single modification. Specifically, the sine chaotic opposition-based learning strategy improves the initial population distribution and enhances early-stage exploration, while the Adaptive Directional Search Mechanism strengthens adaptive exploitation. The stochastic binary perturbation strategy effectively maintains population diversity and prevents premature convergence, and the centroid-based handling mechanism stabilizes the search process by preserving feasibility.

From a behavioral perspective, these mechanisms collectively establish a dynamic search process in which exploration and exploitation are adaptively coordinated across different optimization stages, leading to both rapid convergence and sustained refinement capability.

Furthermore, statistical analyses, including non-parametric significance tests and ranking evaluations, confirm that the observed performance improvements are highly significant and not due to random variation. Overall, IPKO exhibits strong robustness and scalability, demonstrating its effectiveness as a reliable optimization framework for complex benchmark problems.

## 5. Application of the IPKO Algorithm to Engineering Optimization Problems

To further validate the practical applicability and engineering relevance of the proposed IPKO algorithm, this section investigates its performance on two representative real-world optimization problems: cold chain logistics routing and dynamic multi-UAV cooperative path planning. These applications encompass diverse optimization characteristics, including mixed discrete–continuous decision variables, high-dimensional search spaces and strong nonlinearity.

Unlike benchmark test functions, these engineering problems require not only superior objective values but also strict feasibility, solution stability, and robustness under complex constraints. Therefore, they constitute a rigorous testbed for evaluating the practical effectiveness of IPKO. Comparative results demonstrate that IPKO consistently outperforms the baseline PKO and several state-of-the-art metaheuristics in terms of solution quality, convergence behavior, and robustness, highlighting its strong potential for real-world engineering decision support.

### 5.1. Cold Chain Logistics Distribution Optimization

#### 5.1.1. Problem Definition and Dataset

The cold chain logistics routing problem considered in this study is defined as follows. A single distribution center located at coordinates (15,39) dispatches refrigerated vehicles to serve 15 customer demand points. Each vehicle has a maximum load capacity of 5 t and a maximum driving range of 180 km. Each customer is associated with a specific demand, a soft time window, and a hard time window. Vehicles must arrive within the hard time window; otherwise, a large penalty is imposed. If a vehicle arrives earlier than the allowable service time, waiting is permitted until service can begin. During transportation, fresh products deteriorate continuously over time and additionally during unloading operations caused by door opening. Meanwhile, the refrigeration system continuously consumes energy and produces carbon emissions.

The customer data (coordinates, demand, and time windows) are taken from the illustrative example in [44] and are summarized in Table 23. The departure time from the depot is set to 5:00, the average vehicle speed is 45 km/h, and the unloading efficiency is 4 t/h. Other experimental parameters are listed in Table 24.

#### 5.1.2. Mathematical Model

Decision variables: A real vector of length $2N_{c}$ ($N_{c}=15$). The first $N_{c}$ components are sorted in descending order to determine the customer visit sequence; the last $N_{c}$ components are linearly mapped to [1,K] (with K=3 available vehicles) to assign each customer to a vehicle.

Objective function: Minimize the total cost *f*, which consists of fixed cost $F_{1}$, transportation cost $F_{2}$, cargo damage cost $F_{3}$, refrigeration cost $F_{4}$, transport carbon cost $F_{5}$, refrigeration carbon cost $F_{6}$, time-window penalty $F_{7}$, and penalty terms for load/mileage violations (${\mathrm{limit}}_{1}$, ${\mathrm{limit}}_{2}$):

$$f=F_{1}+F_{2}+F_{3}+F_{4}+F_{5}+F_{6}+F_{7}+{\mathrm{limit}}_{1}+{\mathrm{limit}}_{2}.$$

Each component is defined as follows:

- Fixed cost: $F_{1}=\sum_{j=1}^{K}\delta_{j}\cdot p_{1}$, where $\delta_{j}=1$ if vehicle *j* is used.
- Transportation cost: $F_{2}=\sum_{j=1}^{K}D_{j}\cdot p_{2}$, where $D_{j}$ is the total distance (km) traveled by vehicle *j*.
- Cargo damage cost:
  $$F_{3}=p_{3}\sum_{j=1}^{K}\sum_{i\in {\mathrm{path}}_{j}}Q_{\mathrm{now}}\left({\mathrm{arf}}_{1}\cdot \left(T_{i}-t_{0}\right)+{\mathrm{arf}}_{2}\cdot \frac{q_{i}}{\omega }\right),$$
  where $Q_{\mathrm{now}}$ is the current load before serving customer *i* (t), $T_{i}$ is the arrival time (h), $q_{i}$ is the demand (t), and ω=4 t/h.
- Refrigeration cost: The refrigeration system is driven by the vehicle engine. The instantaneous cooling powers (in watts) are first calculated and then converted to kilowatts:
  $$P_{\mathrm{trans}}=\frac{\left(1+\phi \right)\;R\;\sqrt{S_{w}S_{n}}\;\left(T_{w}-T_{n}\right)}{1000}\;\left[\mathrm{kW}\right],\;P_{\mathrm{open}}=\frac{u\;\left(0.54V+3.22\right)\;\left(T_{w}-T_{n}\right)}{1000}\;\left[\mathrm{kW}\right].$$
  The total refrigeration energy consumption (kWh) is:
  $$E_{\mathrm{ref}}=\sum_{j=1}^{K}\left(P_{\mathrm{trans}}\cdot \frac{D_{j}}{v}+\sum_{i\in {\mathrm{path}}_{j}}P_{\mathrm{open}}\cdot \frac{q_{i}}{\omega }\right).$$
  Let SFC be the specific fuel consumption of the refrigeration unit (L/kWh). In this study, SFC=0.28 L/kWh (typical for diesel-driven refrigeration units). The diesel fuel consumed for cooling is ${\mathrm{Fuel}}_{\mathrm{ref}}=\mathrm{SFC}\cdot E_{\mathrm{ref}}$. Then the refrigeration cost is:
  $$F_{4}=p_{\mathrm{fuel}}\cdot {\mathrm{Fuel}}_{\mathrm{ref}},$$
  where $p_{\mathrm{fuel}}$ is the diesel price (yuan/L), set to 7.5 yuan/L in this study.
- Transport carbon emission cost:
  $$F_{5}=p_{5}e_{1}\sum_{j=1}^{K}\sum_{i\in {\mathrm{path}}_{j}}\left(\rho_{1}+\frac{\rho_{2}-\rho_{1}}{Q_{2}}Q_{\mathrm{now}}\right)d_{i,i+1},$$
  where $d_{i,i+1}$ is the distance between consecutive customers (or depot). The term in parentheses is the fuel consumption rate (L/km), and $e_{1}$ (kg/L) is the ${\mathrm{CO}}_{2}$ emission factor of diesel.
- Refrigeration carbon emission cost: The carbon emissions from refrigeration are proportional to the diesel consumed:
  $$F_{6}=p_{5}\cdot e_{2}\cdot {\mathrm{Fuel}}_{\mathrm{ref}},$$
  where $e_{2}$ (kg/L) is the ${\mathrm{CO}}_{2}$ emission factor of diesel used for refrigeration. For consistency with the transport carbon factor, we set $e_{2}=e_{1}=2.63$ kg/L.
- Time-window penalty:
  $$F_{7}=\sum_{i=1}^{N_{c}}\left\{\begin{array}{cc} 10^{5} & T_{i}<{\mathrm{hard}\_\mathrm{start}}_{i}\;\mathrm{or}\;T_{i}>{\mathrm{hard}\_\mathrm{end}}_{i}\\ 100\times ({\mathrm{soft}\_\mathrm{start}}_{i}-T_{i}) & {\mathrm{hard}\_\mathrm{start}}_{i}\leq T_{i}<{\mathrm{soft}\_\mathrm{start}}_{i}\\ 100\times (T_{i}-{\mathrm{soft}\_\mathrm{end}}_{i}) & {\mathrm{soft}\_\mathrm{end}}_{i}<T_{i}\leq {\mathrm{hard}\_\mathrm{end}}_{i}\\ 0 & \mathrm{otherwise}. \end{array}\right.$$
- Constraint violation penalties:
  $${\mathrm{limit}}_{1}=\sum_{j=1}^{K}\max\left(0,\sum_{i\in {\mathrm{path}}_{j}}q_{i}-Z_{\max}\right)\times 10^{7},\;{\mathrm{limit}}_{2}=\sum_{j=1}^{K}\max\left(0,D_{j}-L_{\max}\right)\times 10^{7}.$$

Constraints:

(28) $$\begin{array}{cc} \sum_{i\in {\mathrm{path}}_{j}}q_{i} & \leq Z_{\max}=5\;\left(\forall j\right), \end{array}$$

(29) $$\begin{array}{cc} D_{j} & \leq L_{\max}=180\;\left(\forall j\right), \end{array}$$

(30) $$\begin{array}{cc} {\mathrm{hard}\_\mathrm{start}}_{i}\leq T_{i} & \leq {\mathrm{hard}\_\mathrm{end}}_{i}\;\left(\forall i\right). \end{array}$$

The formulated optimization problem reflects several practical characteristics of real-world cold chain logistics systems. Vehicle load and mileage constraints ensure transportation feasibility and operational safety. Soft and hard time windows are introduced to model customer service satisfaction and perishability requirements of fresh products. Cargo deterioration and refrigeration energy consumption are explicitly incorporated to capture the time-sensitive nature of refrigerated transportation. Furthermore, carbon-emission-related costs are considered to reflect low-carbon logistics requirements and environmental sustainability objectives. All constraints are enforced during optimization.

#### 5.1.3. Experimental Setup

IPKO is compared with the original Pied Kingfisher Optimizer (PKO), Particle Swarm Optimization (PSO), Grey Wolf Optimizer (GWO), and Genetic Algorithm (GA). All algorithms use the same population size (N=80) and maximum number of iterations ($T_{\max}=100$). Each algorithm is run independently 30 times. The same real-number encoding and penalty-based constraint handling are used for all competitors. Standard parameter settings are adopted for each baseline algorithm.

#### 5.1.4. Results and Statistical Analysis

As shown in Table 25, the proposed IPKO algorithm exhibits superior overall performance in solving the cold chain logistics problem. Although the best solution of GWO is slightly better, IPKO achieves the lowest mean total cost (3984.12) across 30 independent runs, indicating its excellent optimization accuracy. More importantly, IPKO obtains the smallest standard deviation (21.79), which is less than half of that of GWO (44.11) and PKO (45.92). This demonstrates that IPKO has significantly higher stability and robustness, which is crucial for practical cold chain logistics applications where consistent cost control is required. The Friedman test (*p* < 0.001) confirms that the performance differences among the algorithms are statistically significant, and IPKO achieves the best Friedman rank of 1.67, further verifying its overall superiority over the compared algorithms.

Table 26 presents the Holm post-hoc test, comparing each algorithm against IPKO. The null hypothesis is rejected for GA, PSO, and PKO, indicating that IPKO is statistically significantly better than these three algorithms at the significance level α=0.05 after Holm correction. For GWO, however, the adjusted *p*-value is 0.1359, which is greater than 0.05, so the difference between IPKO and GWO is not statistically significant at the chosen level. Although the difference between IPKO and GWO is not statistically significant, IPKO achieves a lower mean total cost and a much smaller standard deviation, demonstrating superior stability and practical reliability for cold chain logistics optimization.

The best solution found by IPKO has a total cost of 3943.21 yuan, with no time-window violation (zero penalty). The detailed vehicle routes are:

- Vehicle 1: 0 → 10 → 11 → 6 → 9 → 12 → 0, load = 3.40 t, distance = 53.06 km, finish time = 7.21 h.
- Vehicle 2: 0 → 2 → 1 → 15 → 8 → 4 → 7 → 0, load = 3.30 t, distance = 48.40 km, finish time = 6.90 h.
- Vehicle 3: 0 → 3 → 14 → 13 → 5 → 0, load = 3.60 t, distance = 49.42 km, finish time = 7.27 h.

These results confirm that IPKO effectively balances economic and environmental objectives while satisfying all hard constraints.

#### 5.1.5. Discussion and Practical Implications

From an engineering optimization perspective, the superior performance of IPKO can be attributed to the synergistic integration of multiple search strategies. The sine chaotic opposition-based learning mechanism enhances population diversity during initialization, thereby improving the probability of discovering high-quality feasible regions in the early search stage. The adaptive directional search mechanism and stochastic binary perturbation further strengthen the balance between global exploration and local exploitation. Moreover, the centroid-based adaptive boundary handling strategy effectively guides infeasible individuals back toward feasible regions while preserving population diversity under strict operational constraints.

In practical cold chain logistics operations, even modest percentage improvements in routing cost can translate into substantial economic savings, reduced carbon emissions, and improved service reliability. Therefore, the demonstrated performance of IPKO highlights its strong potential as a robust and reliable decision-support tool for real-world logistics optimization.

### 5.2. Application of the IPKO Algorithm in Dynamic Multi-UAV Cooperative Path Planning

#### 5.2.1. Dynamic Multi-UAV Environment and Problem Description

To further evaluate the effectiveness and scalability of the proposed IPKO algorithm in high-dimensional and constrained optimization scenarios, a three-dimensional dynamic multi-UAV cooperative path-planning problem is constructed as a benchmark test case. Compared with static single-UAV planning, this scenario introduces coupled spatial-temporal constraints, including terrain avoidance, dynamic obstacle interaction, and inter-UAV collision avoidance, which significantly increases the nonlinearity and dimensionality of the optimization problem [45,46,47,48].

##### Decision Variables and Search Space

A fleet of M=3 UAVs is deployed, each assigned a distinct start position $S_{m}=\left(x_{m}^{s},y_{m}^{s},z_{m}^{s}\right)$ and target position $G_{m}=\left(x_{m}^{g},y_{m}^{g},z_{m}^{g}\right)$, m=1,2,3. The trajectory of each UAV is parameterized by N=4 intermediate three-dimensional control points. Four control points are selected as a compromise between trajectory flexibility and optimization dimensionality. The complete decision vector $x\in R^{D}$ of dimension D=3MN=36 is defined as:

$$x={\left[p_{1,1}^{x},\ldots ,p_{1,N}^{x},p_{1,1}^{y},\ldots ,p_{1,N}^{y},p_{1,1}^{z},\ldots ,p_{1,N}^{z},\;\ldots \;,p_{M,1}^{x},\ldots ,p_{M,N}^{z}\right]}^{⊤},$$

where

$$p_{m,k}=\left(p_{m,k}^{x},p_{m,k}^{y},p_{m,k}^{z}\right)$$

denotes the *k*-th control point of UAV *m*. The search space is bounded by the operational region [20,200]×[20,200]×[20,200], i.e., each coordinate component lies in [20,200].

##### Environment Modeling

The terrain is generated using a composite nonlinear elevation model:

$$Z_{\mathrm{terrain}}\left(x,y\right)=30\sin\left(0.03x\right)\cos\left(0.03y\right)+\sum_{i=1}^{10}50\exp\;\left(-\frac{{\left(x-c_{x,i}\right)}^{2}+{\left(y-c_{y,i}\right)}^{2}}{1500}\right),$$

where $\left(c_{x,i},c_{y,i}\right)$ are random peak centers. This produces irregular mountainous structures. Terrain clearance requires that at any point (x,y,z) on a UAV trajectory,

$$z\geq Z_{\mathrm{terrain}}\left(x,y\right).$$

Five dynamic obstacles are introduced. Each obstacle *k* (k=1,…,5) has a time-varying position $O_{k}\left(t\right)=\left(x_{k}\left(t\right),y_{k}\left(t\right),z_{k}\left(t\right)\right)$ following a linear motion model:

$$O_{k}\left(t\right)=O_{k}\left(0\right)+v_{k}t,\;t=1,2,\ldots ,T,$$

with T=200 discrete time steps, initial position $O_{k}\left(0\right)$ uniformly random in ${\left[0,200\right]}^{2}\times \left\{100\right\}$, and velocity $v_{k}∼N\left(0,0.3^{2}\right)$ in *x* and *y* directions (zero vertical speed). Each obstacle has a threat radius $r_{k}\in \left[8,13\right]$. A UAV is considered at risk when its distance to the obstacle center is less than $r_{k}$.

Cooperative safety requires that at any time step *t*, the Euclidean distance between any two UAVs *i* and *j* satisfies

$$d_{ij}\left(t\right)\geq D_{\mathrm{safe}},$$

where $D_{\mathrm{safe}}=10$ is the minimum separation distance. All constraints are handled via penalty functions in the objective.

##### Altitude Operational Constraints

In addition to terrain clearance, each UAV must satisfy operational altitude limits throughout the mission:

$$z_{\min}\leq z_{m}\left(t\right)\leq z_{\max},\;\forall m=1,\ldots ,M,\;t=1,\ldots ,T,$$

where $z_{\min}=20$ and $z_{\max}=200$. Although all control points are constrained within the interval [20,200], the altitude limits are additionally verified on all sampled trajectory points to account for possible spline interpolation overshoot.

##### Dynamic Environment Assumptions

The environment is dynamic but deterministic. The motion patterns of dynamic obstacles are assumed to be predictable, i.e., their positions as functions of time are provided in advance. This corresponds to scenarios where obstacles follow known schedules (e.g., airport ground vehicles, ships on shipping lanes) or where their future positions can be estimated with sufficient accuracy. The UAVs are assumed to operate synchronously with discrete time steps, and the planning is performed offline before mission execution.

The resulting formulation constitutes a nonlinear constrained optimization problem with coupled spatial-temporal constraints, making it considerably more challenging and practically relevant than conventional static benchmark path-planning problems.

#### 5.2.2. Path Representation and Objective Function Formulation

For each UAV *m*, a smooth trajectory is generated by cubic spline interpolation through the sequence: start point $S_{m}$, control points $p_{m,1},\ldots ,p_{m,N}$, and goal point $G_{m}$. The interpolation is performed over T=200 equally spaced time steps, yielding a continuous spline trajectory sampled at T=200 discrete time steps. The position of UAV *m* at time *t* is denoted $q_{m}\left(t\right)\in R^{3}$.

The optimization problem is formulated as a single-objective minimization with penalty functions for constraint violations:

$$\min_{x}\;f\left(x\right)=L+\alpha_{1}P_{\mathrm{terrain}}+\alpha_{2}P_{\mathrm{obs}}+\alpha_{3}P_{\mathrm{UAV}}+\alpha_{4}P_{\mathrm{coop}}+\alpha_{5}P_{\mathrm{smooth}},$$

where the weight coefficients are set to

$$\alpha_{1}=80,\;\alpha_{2}=40,\;\alpha_{3}=20,\;\alpha_{4}=10,\;\alpha_{5}=1,$$

prioritizing safety and feasibility over pure path efficiency.

##### Path Length Term *L*

The total flight path length normalized by the straight-line distance from start to goal:

$$L=\sum_{m=1}^{M}\frac{\sum_{t=1}^{T-1}{∥q_{m}\left(t+1\right)-q_{m}\left(t\right)∥}_{2}}{∥G_{m}-S_{m}{∥}_{2}}.$$

##### Terrain Clearance Penalty $P_{\mathrm{terrain}}$

Violations of the terrain constraint are penalized quadratically:

$$P_{\mathrm{terrain}}=\sum_{m=1}^{M}\sum_{t=1}^{T}\max\;{\left(0,\;Z_{\mathrm{terrain}}\left(x_{m}\left(t\right),y_{m}\left(t\right)\right)-z_{m}\left(t\right)\right)}^{2},$$

where $\left(x_{m}\left(t\right),y_{m}\left(t\right),z_{m}\left(t\right)\right)$ are the coordinates of $q_{m}\left(t\right)$. $Z_{\mathrm{terrain}}\left(\cdot ,\cdot \right)$ is evaluated via bilinear interpolation on the terrain grid.

##### Dynamic Obstacle Collision Penalty $P_{\mathrm{obs}}$

For each UAV and each dynamic obstacle, a penalty is incurred when the UAV enters the obstacle’s threat radius:

$$P_{\mathrm{obs}}=\sum_{m=1}^{M}\sum_{k=1}^{5}\sum_{t=1}^{T}\max\;{\left(0,\;r_{k}-{∥q_{m}\left(t\right)-O_{k}\left(t\right)∥}_{2}\right)}^{2}.$$

##### Inter-UAV Collision Penalty $P_{\mathrm{UAV}}$

This term enforces the minimum separation distance:

$$P_{\mathrm{UAV}}=\sum_{t=1}^{T}\sum_{i=1}^{M-1}\sum_{j=i+1}^{M}\max\;{\left(0,\;D_{\mathrm{safe}}-{∥q_{i}\left(t\right)-q_{j}\left(t\right)∥}_{2}\right)}^{2}.$$

##### Cooperative Spacing Penalty $P_{\mathrm{coop}}$

To encourage larger inter-UAV distances beyond the strict safety margin, an inverse-distance term is added:

$$P_{\mathrm{coop}}=\sum_{t=1}^{T}\sum_{i=1}^{M-1}\sum_{j=i+1}^{M}\frac{1}{∥q_{i}\left(t\right)-q_{j}{\left(t\right)∥}_{2}+\varepsilon },$$

with $\varepsilon =10^{-3}$ to avoid division by zero. This term prevents unnecessary proximity and improves overall formation safety.

##### Trajectory Smoothness Penalty $P_{\mathrm{smooth}}$

To further suppress excessive turning and abrupt trajectory changes, a second-order difference penalty is introduced:

$$P_{\mathrm{smooth}}=\sum_{m=1}^{M}\sum_{t=2}^{T-1}{∥q_{m}\left(t+1\right)-2q_{m}\left(t\right)+q_{m}\left(t-1\right)∥}_{2}^{2}.$$

This term approximates trajectory curvature and acceleration variation, thereby encouraging smoother and more energy-efficient flight paths that are more suitable for practical UAV operations.

All constraints (terrain, obstacle, inter-UAV separation) are treated as soft constraints via penalties. The weighted sum formulation allows the optimizer to balance efficiency against feasibility, with sufficiently high penalty coefficients to ensure that feasible solutions are strongly favored.

#### 5.2.3. Experimental Results and Analysis

This subsection evaluates IPKO against PKO, IVY [49], HO [50], and WOA on the dynamic multi-UAV path-planning problem. All algorithms use population size 30, 100 iterations, and 30 independent runs. To ensure fairness, all algorithms share identical environment settings, cost function definitions, and stopping criteria.

- Quantitative Performance Evaluation

Table 27 summarizes the mean and standard deviation of the fitness values obtained by all compared algorithms. Among them, IPKO achieves the lowest mean fitness value (49.24), outperforming PKO (52.09), IVY (52.21), HO (57.10), and WOA (58.67), with relative improvements ranging from 5.47% to 16.07%. In addition, IPKO exhibits a relatively low standard deviation (3.10), indicating stable optimization behavior and good robustness across independent runs.

To further verify whether these performance differences are statistically significant, the non-parametric Friedman test was first conducted, since it does not rely on assumptions of normality or homogeneity of variance. The obtained *p*-value ($2.13\times 10^{-10}$) strongly rejects the null hypothesis that all algorithms exhibit equivalent optimization performance. The corresponding average rankings (lower values indicate better performance) are: IPKO (1.73), PKO (2.57), IVY (2.57), WOA (4.03), and HO (4.10).

Table 28 summarizes the Holm post-hoc procedure results. The Holm-adjusted *p*-values indicate that IPKO significantly outperforms HO and WOA (adjusted p<0.05), whereas the performance differences between IPKO and PKO/IVY are not statistically significant at the 5% significance level. Nevertheless, IPKO still achieves the best average ranking and the lowest mean fitness value among all compared methods.

These results demonstrate that the proposed IPKO maintains strong competitiveness against state-of-the-art optimizers while exhibiting statistically significant superiority over inferior-performing algorithms. Such improvements can be attributed to the collaborative effect of the proposed enhancement strategies, including chaotic initialization, adaptive directional updates, stochastic information fusion, and centroid-based adaptive boundary handling. Overall, the statistical analyses further confirm the reliability and robustness of the proposed IPKO in practical UAV path-planning optimization tasks.

B. Convergence Behavior Analysis

**Figure 12:**
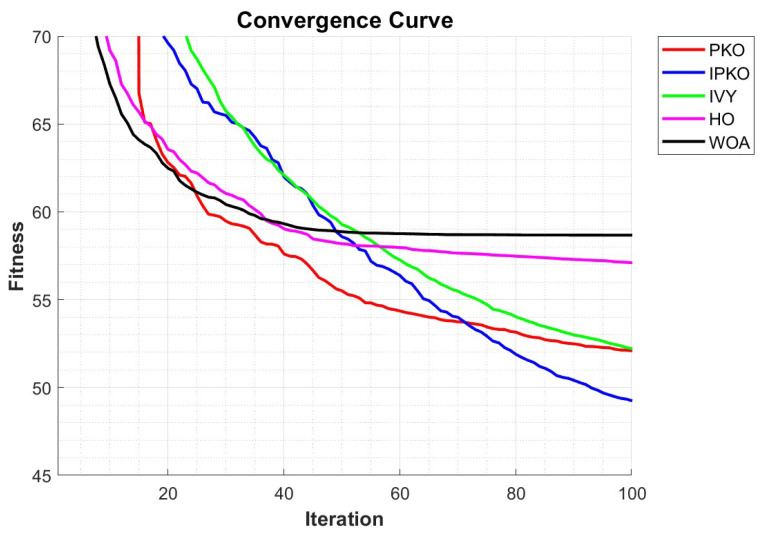
Convergence curves of all compared algorithms on the UAV path-planning problem.

Figure 12 shows the convergence curves. IPKO achieves faster fitness reduction in early iterations and continues refining solutions in later stages, while baseline algorithms exhibit premature convergence or stagnation. The smoother convergence profile reflects improved algorithmic stability.

C. Three-Dimensional Path Visualization

**Figure 13:**
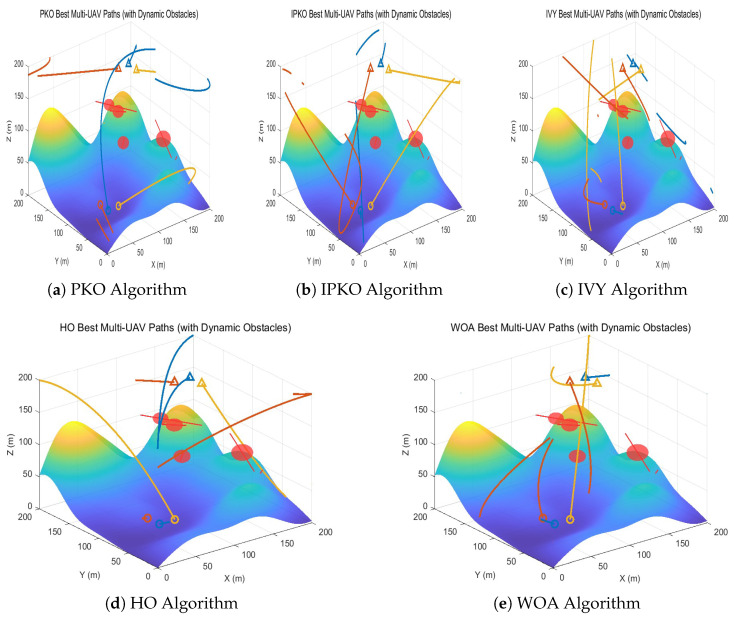
Best 3D trajectories obtained by each algorithm. IPKO produces smoother paths with better terrain and obstacle avoidance.

Figure 13 visualizes the best paths from each algorithm. Red circles denote dynamic obstacles. IPKO generates smoother trajectories with gradual altitude changes, effective terrain adaptation, and consistent safety margins. Inter-UAV distances remain above the safety threshold throughout.

In contrast, PKO produces unnecessarily high altitudes; IVY and WOA exhibit sharp turns and altitude fluctuations; HO maintains smoothness but occasionally violates obstacle constraints.

#### 5.2.4. Discussion and Practical Implications

The experimental results demonstrate that IPKO consistently outperforms competing algorithms across all metrics, with improvements attributable to its synergistic enhancement mechanisms. Chaotic initialization enables broader exploration, adaptive updates maintain population diversity for continued refinement, and centroid-based handling ensures path feasibility in constrained environments. These advantages translate directly to practical benefits: smoother trajectories reduce mechanical stress and energy consumption, consistent safety margins enhance operational reliability in dynamic settings, and robust performance across independent runs supports real-world deployment in safety-critical applications such as search-and-rescue and infrastructure inspection. While IPKO incurs slightly higher computational cost, this trade-off is justified by substantial gains in solution quality and constraint compliance for complex multi-UAV path-planning scenarios.

Although this study primarily compares IPKO with representative population-based metaheuristic optimizers, future work will further include comparisons with domain-specific UAV path-planning approaches, such as RRT*-based planners, A*-based methods, and hybrid PSO trajectory optimization frameworks, to provide a more comprehensive engineering evaluation.

### 5.3. Engineering Application Summary

The engineering case studies on cold chain logistics and multi-UAV path planning were deliberately selected to represent two fundamentally different optimization paradigms: discrete combinatorial optimization with strict operational constraints and continuous path planning in dynamic environments. Despite these structural differences, the experimental results exhibit consistent performance patterns, demonstrating the strong generalizability of the proposed IPKO framework.

Across both applications, IPKO consistently achieves a favorable balance among multiple competing objectives, including cost efficiency, constraint satisfaction, and solution stability. This performance can be directly attributed to its hybrid enhancement mechanisms. In particular, the centroid-based handling strategy plays a crucial role in maintaining feasibility by guiding infeasible solutions back toward the population center, which effectively avoids disruptive boundary truncation. This mechanism proves equally effective in preserving temperature constraints in cold chain routing and ensuring collision-free trajectories in UAV navigation, highlighting its adaptability across different constraint types.

Moreover, the performance advantage of IPKO over the baseline PKO becomes more pronounced in the dynamic UAV scenario. This observation suggests that the adaptive directional search and stochastic perturbation strategies enable IPKO to better respond to dynamically changing search landscapes by continuously injecting directional diversity and preventing premature convergence.

Overall, these cross-domain results confirm that IPKO is not merely a problem-specific optimizer but a robust and general-purpose metaheuristic. The strong consistency between algorithmic design and observed performance supports the underlying design principle that synergistic enhancement mechanisms—combining diversity preservation, adaptive guidance, and feasibility control—can effectively generalize across heterogeneous optimization problems.

## 6. Conclusions and Future Work

This paper presents a biomimetic enhancement of the pied kingfisher optimizer. The Improved Pied Kingfisher Optimizer (IPKO) overcomes the phase-specific limitations of the original PKO by integrating four complementary strategies: sine chaotic opposition-based initialization, adaptive directional search, stochastic binary perturbation-based information fusion, and centroid-based adaptive boundary handling. These mechanisms collectively enhance population diversity, search guidance, local optima escape, and constraint feasibility.

Extensive experiments on the CEC2017 benchmark suite and two engineering optimization problems demonstrate that IPKO consistently outperforms eleven state-of-the-art algorithms, achieving the best overall ranking with statistically significant improvements. Ablation studies further verify the effectiveness of each component. In practical applications, IPKO achieves notable improvements in solution quality, particularly in cold chain logistics and dynamic multi-UAV cooperative path planning, indicating its applicability to real-world optimization scenarios.

Overall, the results demonstrate that IPKO provides a reliable and efficient optimization method for solving complex, high-dimensional, and constrained problems.

Future work will focus on extending IPKO to large-scale and dynamic optimization environments. Additionally, adaptive parameter control strategies and more advanced information interaction mechanisms will be explored to further enhance scalability and convergence performance. Future research will also investigate hybrid frameworks and real-time optimization scenarios to improve the applicability of IPKO in more complex and uncertain environments.

## Figures and Tables

**Table 1:** Illustrative interaction of *B*, θ, and *V* (assuming $X\left(r_{1},:\right)-X\left(i,:\right)=\left(1,0\right)$ and $X\left(r_{1},:\right)=\left(1,0\right)$ for simplicity).

*B*	θ	*V*	Approximate Move Direction and Magnitude
0.2 (early)	0°	2	Small radial step toward peer
0.2 (early)	90°	5	Small lateral step (sine dominated)
1.8 (late)	0°	2	Large radial step toward peer (controlled)
1.8 (late)	0°	5	Very large radial jump (aggressive escape)
1.8 (late)	90°	2	Large lateral move, little radial change

**Table 2:** Comparison between IPKO and representative optimizers used in this study.

Algorithm	Key Strategies	Integration Type	Stage-Specific Design	Adaptive Coordination
WOA, GWO, DE, PKO	Single search mechanism	None	No	No
NRBO, KO, POA, HBA, GBO	Single/dual mechanisms	Simple	No	Partial
L-SHADE [35]	Success-history adaptation, linear population size reduction	Sequential	Partial	Yes
CMA-ES [36]	Covariance matrix adaptation, path length control	Sequential	No	Yes
IPKO (this work)	Chaotic init, OBL, adaptive directional search, stochastic perturbation, centroid-based repair	Phase-deficiency-driven integration	Yes (each strategy targets a specific deficiency of PKO)	Coordinated (stagnation detection + adaptive activation)

**Table 3:** Phase-deficiency-oriented enhancement framework of IPKO.

PKO Phase	Identified Limitation	Proposed Mechanism	Expected Effect
Initialization	Insufficient diversity and uneven distribution	Sine chaotic opposition-based initialization	Improved population diversity and spatial coverage
Exploration	Lack of effective search guidance	Adaptive directional search mechanism	Enhanced search efficiency and guided exploration
Exploitation	Premature convergence to local optima	Stochastic binary perturbation-based information fusion strategy	Improved ability to escape local optima
Constraint handling	Boundary violations	Centroid-based adaptive boundary handling mechanism	Robust feasibility maintenance

**Table 4:** Comparative Results of 12 algorithms on cec2017 test functions (D = 30).

		IPKO	PKO	NRBO	KO	POA	HBA	GBO	WOA	GWO	DE	LSHADE	CMAES
F1	std	**2.70e+03**	4.63e+06	5.02e+09	2.09e+06	5.43e+09	3.16e+06	4.31e+05	3.63e+09	1.55e+09	7.89e+05	2.13e+03	3.45e+04
F1	mean	3.62e+03	3.66e+06	1.48e+10	5.95e+06	2.11e+10	1.57e+06	2.64e+05	5.35e+09	2.69e+09	1.27e+06	**1.97e+03**	2.34e+04
F1	art	2.85e-01	9.69e-02	9.56e-02	4.98e-01	6.93e-02	7.70e-02	1.31e-01	**4.39e-02**	8.96e-02	1.15e-01	1.23e-01	4.64e-01
F3	std	**5.82e+03**	2.84e+04	1.14e+04	9.64e+03	7.02e+03	9.73e+03	6.21e+03	4.98e+04	6.77e+03	2.45e+04	1.43e+04	1.74e+05
F3	mean	2.21e+04	1.38e+05	5.65e+04	5.97e+04	3.96e+04	4.12e+04	**2.15e+04**	2.66e+05	6.45e+04	1.50e+05	8.36e+04	4.09e+05
F3	art	2.85e-01	9.67e-02	9.45e-02	4.45e-01	7.03e-02	7.80e-02	1.29e-01	**3.99e-02**	8.85e-02	1.11e-01	7.18e-02	3.86e-01
F4	std	2.28e+01	1.78e+01	1.05e+03	1.45e+01	1.38e+03	1.58e+01	1.35e+01	2.05e+02	4.81e+01	1.58e+01	1.37e+01	**5.07e-01**
F4	mean	5.00e+02	5.18e+02	2.50e+03	5.44e+02	2.33e+03	4.85e+02	4.95e+02	1.12e+03	5.76e+02	5.17e+02	4.99e+02	**4.23e+02**
F4	art	2.80e-01	9.56e-02	9.29e-02	5.02e-01	7.27e-02	7.95e-02	1.28e-01	**4.00e-02**	8.88e-02	1.13e-01	1.05e-01	3.89e-01
F5	std	2.24e+01	3.31e+01	3.71e+01	2.65e+01	2.51e+01	2.92e+01	4.07e+01	1.99e+01	1.95e+01	**5.54e+00**	1.34e+01	7.45e+01
F5	mean	**5.76e+02**	5.87e+02	8.06e+02	6.94e+02	7.90e+02	6.18e+02	6.31e+02	8.70e+02	6.16e+02	7.04e+02	6.67e+02	6.33e+02
F5	art	3.25e-01	1.26e-01	1.09e-01	4.75e-01	1.03e-01	9.74e-02	1.49e-01	**5.66e-02**	1.21e-01	1.60e-01	9.06e-02	4.46e-01
F6	std	4.72e-01	1.54e+00	1.09e+01	7.88e+00	6.17e+00	7.33e+00	1.80e+01	4.05e+00	6.28e+00	3.86e-02	3.63e-03	**2.08e-03**
F6	mean	**6.00e+02**	6.02e+02	6.70e+02	6.39e+02	6.65e+02	6.24e+02	6.37e+02	6.75e+02	6.13e+02	6.00e+02	6.00e+02	6.00e+02
F6	art	4.92e-01	2.25e-01	1.55e-01	6.02e-01	1.84e-01	1.37e-01	1.87e-01	**9.84e-02**	1.51e-01	1.77e-01	1.43e-01	4.59e-01
F7	std	2.23e+01	4.82e+01	4.06e+01	6.99e+01	7.15e+01	2.27e+01	4.65e+01	6.08e+01	5.46e+01	1.53e+01	1.49e+01	**6.35e+00**
F7	mean	**8.31e+02**	8.89e+02	1.26e+03	1.03e+03	1.26e+03	9.25e+02	9.89e+02	1.33e+03	8.94e+02	9.31e+02	9.15e+02	9.13e+02
F7	art	3.53e-01	1.35e-01	1.12e-01	4.95e-01	1.04e-01	9.54e-02	1.46e-01	**5.69e-02**	1.05e-01	1.32e-01	9.14e-02	4.09e-01
F8	std	1.29e+01	4.07e+01	2.11e+01	2.22e+01	2.54e+01	3.11e+01	3.78e+01	3.08e+01	2.86e+01	**7.41e+00**	2.08e+01	5.85e+01
F8	mean	8.69e+02	8.94e+02	1.08e+03	9.53e+02	9.80e+02	9.06e+02	9.31e+02	1.08e+03	9.12e+02	9.99e+02	9.79e+02	**8.58e+02**
F8	art	3.36e-01	1.44e-01	1.13e-01	4.85e-01	1.06e-01	9.65e-02	1.45e-01	**5.59e-02**	1.07e-01	1.28e-01	8.89e-02	4.13e-01
F9	std	2.11e+02	2.38e+02	1.65e+03	1.64e+03	4.13e+02	5.65e+02	6.74e+02	3.08e+03	7.38e+02	7.47e+02	7.75e+00	**1.41e-04**
F9	mean	1.48e+03	1.43e+03	7.03e+03	7.92e+03	5.98e+03	2.88e+03	3.07e+03	1.22e+04	2.15e+03	2.35e+03	9.06e+02	**9.00e+02**
F9	art	3.35e-01	1.31e-01	1.12e-01	5.05e-01	1.05e-01	9.66e-02	1.46e-01	**5.63e-02**	1.05e-01	1.29e-01	1.03e-01	4.12e-01
F10	std	7.04e+02	6.07e+02	5.76e+02	1.92e+03	5.74e+02	1.01e+03	5.05e+02	**2.59e+02**	1.45e+03	2.65e+02	5.41e+02	4.24e+02
F10	mean	**4.20e+03**	5.13e+03	8.05e+03	6.78e+03	5.37e+03	5.30e+03	4.97e+03	8.16e+03	5.04e+03	7.60e+03	8.16e+03	9.11e+03
F10	art	3.64e-01	1.53e-01	1.23e-01	5.52e-01	1.23e-01	1.06e-01	1.65e-01	**6.66e-02**	1.14e-01	1.40e-01	1.01e-01	4.24e-01
F11	std	**3.92e+01**	5.34e+01	6.85e+02	5.28e+01	1.23e+03	7.12e+01	8.98e+01	3.75e+03	1.25e+03	1.18e+02	4.83e+01	7.80e+03
F11	mean	**1.18e+03**	1.45e+03	2.88e+03	1.33e+03	2.38e+03	1.35e+03	1.27e+03	8.72e+03	2.83e+03	1.75e+03	1.25e+03	1.12e+04
F11	art	3.02e-01	1.12e-01	**1.01e-01**	2.07e+00	4.72e-01	4.23e-01	6.77e-01	2.26e-01	2.57e-01	7.49e-01	1.29e-01	2.10e+00
F12	std	**4.21e+05**	9.95e+05	4.57e+08	7.45e+06	1.29e+09	6.98e+05	6.21e+05	3.24e+08	1.00e+08	2.80e+07	4.59e+05	7.74e+06
F12	mean	6.50e+05	2.22e+06	1.14e+09	1.20e+07	9.48e+08	1.18e+06	1.00e+06	5.65e+08	9.05e+07	4.83e+07	**3.28e+05**	7.92e+06
F12	art	1.76e+00	6.40e-01	4.67e-01	3.18e+00	5.10e-01	4.90e-01	6.64e-01	**1.94e-01**	3.29e-01	7.31e-01	4.53e-01	2.02e+00
F13	std	**4.66e+03**	3.85e+05	2.31e+08	4.98e+04	1.07e+07	2.13e+04	2.18e+04	1.71e+07	4.42e+05	2.31e+06	1.75e+04	4.31e+06
F13	mean	**6.53e+03**	2.67e+05	3.48e+08	1.00e+05	1.06e+07	4.66e+04	1.92e+04	1.36e+07	4.12e+05	3.68e+06	4.68e+04	4.71e+06
F13	art	2.21e+00	6.06e-01	6.01e-01	3.39e+00	5.22e-01	4.83e-01	7.40e-01	**2.63e-01**	3.95e-01	9.40e-01	3.91e-01	1.93e+00
F14	std	2.26e+04	1.99e+05	1.05e+05	2.41e+05	6.59e+03	4.83e+04	5.33e+03	8.26e+05	1.02e+06	2.94e+05	**7.57e+02**	2.84e+05
F14	mean	1.49e+04	1.14e+05	1.17e+05	3.64e+05	1.00e+04	5.78e+04	7.20e+03	8.75e+05	1.23e+06	3.75e+05	**2.36e+03**	7.01e+05
F14	art	2.18e+00	7.94e-01	7.25e-01	3.32e+00	8.01e-01	6.67e-01	8.97e-01	**3.73e-01**	5.28e-01	1.13e+00	6.07e-01	2.24e+00
F15	std	**5.86e+02**	1.26e+04	4.93e+05	5.48e+03	2.56e+04	1.20e+04	1.82e+04	6.78e+05	1.15e+06	6.91e+05	5.29e+03	5.29e+06
F15	mean	**3.08e+03**	2.81e+04	5.02e+05	1.64e+04	5.61e+04	2.13e+04	2.00e+04	2.50e+06	6.08e+05	6.55e+05	8.82e+03	9.50e+06
F15	art	2.04e+00	5.53e-01	5.07e-01	2.00e+00	8.03e-02	8.30e-02	1.33e-01	**4.46e-02**	9.35e-02	1.18e-01	7.86e-02	3.90e-01
F16	std	**1.09e+02**	2.10e+02	4.81e+02	4.41e+02	2.07e+02	7.92e+02	3.44e+02	6.23e+02	2.48e+02	1.12e+02	3.50e+02	3.86e+02
F16	mean	**2.31e+03**	2.74e+03	3.59e+03	3.33e+03	3.40e+03	2.99e+03	2.60e+03	4.29e+03	2.48e+03	2.97e+03	2.81e+03	2.72e+03
F17	std	**6.72e+01**	1.19e+02	3.21e+02	2.55e+02	1.56e+02	8.47e+01	7.14e+01	1.76e+02	1.70e+02	1.39e+02	1.84e+02	1.57e+02
F17	mean	**1.82e+03**	2.11e+03	2.53e+03	2.60e+03	2.37e+03	2.14e+03	2.46e+03	2.58e+03	2.04e+03	2.22e+03	2.11e+03	2.34e+03
F17	art	4.24e-01	1.87e-01	1.39e-01	5.23e-01	1.60e-01	1.24e-01	1.75e-01	**8.45e-02**	1.33e-01	1.58e-01	1.18e-01	4.37e-01
F18	std	6.30e+04	1.33e+06	7.61e+05	7.78e+05	2.85e+05	1.80e+05	2.07e+05	1.77e+07	6.68e+04	2.21e+06	**4.93e+04**	4.80e+06
F18	mean	**8.88e+04**	1.49e+06	1.25e+06	1.00e+06	4.15e+05	3.86e+05	2.62e+05	2.98e+07	1.75e+05	3.52e+06	3.13e+05	5.51e+06
F18	art	3.22e-01	1.22e-01	1.07e-01	4.76e-01	9.63e-02	9.12e-02	1.42e-01	**5.28e-02**	1.01e-01	1.24e-01	8.44e-02	4.01e-01
F19	std	**1.79e+03**	3.03e+04	9.95e+06	8.15e+05	7.05e+05	4.02e+03	6.79e+03	4.69e+07	1.18e+06	3.98e+05	4.25e+03	1.17e+06
F19	mean	**4.08e+03**	3.59e+04	9.15e+06	7.85e+05	7.79e+05	5.85e+03	1.19e+04	4.14e+07	7.06e+05	4.72e+05	7.98e+03	1.51e+06
F19	art	9.62e-01	5.44e-01	3.18e-01	6.97e-01	5.18e-01	3.04e-01	3.56e-01	**2.64e-01**	3.13e-01	3.39e-01	3.03e-01	6.30e-01
F20	std	8.73e+01	8.94e+01	2.41e+02	1.23e+02	1.34e+02	2.07e+02	1.87e+02	1.90e+02	2.15e+02	**7.42e+01**	1.12e+02	2.14e+02
F20	mean	**2.27e+03**	2.44e+03	2.87e+03	2.38e+03	2.50e+03	2.58e+03	2.43e+03	3.08e+03	2.57e+03	2.50e+03	2.48e+03	2.67e+03
F20	art	4.39e-01	1.95e-01	1.43e-01	5.18e-01	1.68e-01	1.27e-01	1.80e-01	**8.80e-02**	1.38e-01	1.65e-01	1.22e-01	4.45e-01
F21	std	**9.88e+00**	1.68e+01	7.17e+01	5.10e+01	5.86e+01	5.63e+01	2.64e+01	6.84e+01	1.76e+01	1.47e+01	2.09e+01	6.31e+01
F21	mean	**2.35e+03**	2.37e+03	2.61e+03	2.44e+03	2.56e+03	2.41e+03	2.43e+03	2.64e+03	2.40e+03	2.50e+03	2.46e+03	2.42e+03
F21	art	5.42e-01	2.66e-01	1.78e-01	5.74e-01	2.42e-01	1.65e-01	2.15e-01	**1.25e-01**	1.73e-01	1.96e-01	1.58e-01	4.80e-01
F22	std	**5.03e-02**	1.98e+03	1.80e+03	2.11e+01	1.80e+03	2.22e+03	2.20e+03	3.00e+03	1.35e+03	2.28e+03	1.10e+00	2.88e+02
F22	mean	**2.30e+03**	5.67e+03	5.41e+03	2.33e+03	6.05e+03	3.31e+03	3.29e+03	7.94e+03	5.06e+03	7.03e+03	2.30e+03	1.02e+04
F22	art	6.01e-01	3.05e-01	2.00e-01	6.09e-01	2.76e-01	1.82e-01	2.33e-01	**1.42e-01**	1.92e-01	2.19e-01	2.05e-01	5.00e-01
F23	std	2.59e+01	2.22e+01	5.39e+01	4.04e+01	3.56e+01	1.12e+02	3.44e+01	1.51e+02	5.07e+01	**1.86e+01**	2.10e+01	5.28e+01
F23	mean	**2.71e+03**	2.75e+03	3.04e+03	2.87e+03	3.03e+03	2.83e+03	2.80e+03	3.13e+03	2.79e+03	2.84e+03	2.82e+03	2.74e+03
F23	art	6.52e-01	3.40e-01	2.17e-01	6.41e-01	3.12e-01	2.00e-01	2.49e-01	**1.61e-01**	2.09e-01	2.34e-01	1.94e-01	5.22e-01
F24	std	1.30e+01	1.36e+01	4.37e+01	4.76e+01	3.91e+01	1.88e+02	5.71e+01	1.00e+02	5.19e+01	9.81e+00	**4.56e+00**	8.09e+01
F24	mean	**2.88e+03**	2.92e+03	3.19e+03	2.95e+03	3.17e+03	3.06e+03	2.97e+03	3.26e+03	3.00e+03	3.04e+03	2.99e+03	2.90e+03
F24	art	7.09e-01	3.75e-01	2.33e-01	6.42e-01	3.50e-01	2.20e-01	2.70e-01	**1.80e-01**	2.30e-01	2.52e-01	2.16e-01	5.43e-01
F25	std	9.72e+00	5.08e+00	6.40e+01	1.13e+01	1.18e+02	1.52e+01	2.11e+01	1.10e+02	3.72e+01	3.35e+00	7.76e-01	**1.05e-01**
F25	mean	2.90e+03	2.92e+03	3.36e+03	2.97e+03	3.30e+03	2.90e+03	2.92e+03	3.26e+03	2.99e+03	2.89e+03	2.89e+03	**2.88e+03**
F25	art	6.40e-01	3.28e-01	2.09e-01	5.88e-01	3.02e-01	1.96e-01	2.45e-01	**1.55e-01**	2.04e-01	2.31e-01	2.20e-01	5.10e-01
F26	std	3.08e+02	3.74e+02	1.26e+03	2.07e+03	1.88e+03	9.26e+02	6.29e+02	2.22e+03	2.00e+02	**1.06e+02**	2.36e+02	6.94e+02
F26	mean	4.59e+03	4.69e+03	8.16e+03	4.40e+03	6.72e+03	**4.17e+03**	5.46e+03	7.97e+03	4.84e+03	5.54e+03	5.30e+03	4.49e+03
F26	art	7.71e-01	4.19e-01	2.55e-01	6.65e-01	3.87e-01	2.41e-01	2.90e-01	**2.01e-01**	2.49e-01	2.76e-01	2.35e-01	5.63e-01
F27	std	1.55e+01	1.01e+01	8.81e+01	3.19e+01	6.86e+01	5.01e+02	1.37e+01	1.21e+02	2.16e+01	4.32e+00	9.09e+00	**5.63e-05**
F27	mean	3.22e+03	3.22e+03	3.44e+03	3.36e+03	3.39e+03	3.65e+03	3.25e+03	3.46e+03	3.26e+03	3.23e+03	3.22e+03	**3.20e+03**
F27	art	8.50e-01	4.73e-01	2.82e-01	7.00e-01	4.44e-01	2.66e-01	3.17e-01	**2.27e-01**	2.76e-01	3.01e-01	2.75e-01	5.92e-01
F28	std	2.44e+01	1.19e+01	4.77e+02	1.13e+01	2.84e+02	3.30e+01	2.45e+01	6.59e+01	7.66e+01	1.21e+01	2.07e+01	**6.12e-05**
F28	mean	3.24e+03	3.29e+03	4.13e+03	3.33e+03	3.94e+03	3.28e+03	3.25e+03	3.58e+03	3.49e+03	3.30e+03	**3.23e+03**	3.30e+03
F28	art	7.62e-01	4.11e-01	2.52e-01	1.34e+00	4.25e-01	2.44e-01	2.95e-01	**1.95e-01**	2.47e-01	2.76e-01	2.77e-01	5.71e-01
F29	std	9.68e+01	1.15e+02	2.79e+02	1.43e+02	3.58e+02	6.73e+02	4.67e+02	5.95e+02	2.50e+02	**6.43e+01**	1.44e+02	1.80e+02
F29	mean	**3.59e+03**	3.86e+03	4.81e+03	4.22e+03	4.52e+03	4.60e+03	4.43e+03	5.90e+03	4.14e+03	4.15e+03	3.91e+03	4.00e+03
F29	art	2.04e+00	1.47e+00	4.33e-01	6.78e-01	2.84e-01	1.87e-01	2.38e-01	**1.44e-01**	1.92e-01	2.18e-01	1.78e-01	4.99e-01
F30	std	1.88e+04	4.33e+04	1.80e+07	2.66e+06	3.58e+06	2.38e+05	**9.11e+03**	1.54e+07	8.09e+06	1.49e+05	9.53e+03	1.37e+06
F30	mean	2.18e+04	9.19e+04	5.66e+07	3.85e+06	9.67e+06	1.34e+05	**1.36e+04**	3.62e+07	6.03e+06	3.36e+05	3.17e+04	1.89e+06
F30	art	1.15e+00	6.73e-01	3.78e-01	7.94e-01	6.51e-01	3.71e-01	4.22e-01	**3.27e-01**	3.75e-01	4.01e-01	3.72e-01	7.16e-01

Note: The best results in each table are highlighted in boldface.

**Table 5:** Comparative results of 12 algorithms on cec2017 test functions (D = 10).

		IPKO	PKO	NRBO	KO	POA	HBA	GBO	WOA	GWO	DE	LSHADE	CMAES
F1	std	7.14e+02	6.97e+04	3.66e+08	4.55e+03	7.01e+08	5.26e+03	4.59e+03	4.91e+07	8.05e+06	7.06e+03	**9.25e-03**	4.45e+05
F1	mean	5.99e+02	5.08e+04	6.36e+08	1.68e+04	3.19e+08	6.96e+03	3.60e+03	6.02e+07	5.79e+06	8.80e+03	**1.00e+02**	6.42e+05
F3	std	1.36e-02	1.15e+03	4.32e+02	3.48e+02	1.59e+03	3.32e-04	**5.18e-05**	8.07e+03	4.02e+03	2.65e+03	9.79e-03	2.68e+04
F3	mean	3.00e+02	2.26e+03	1.65e+03	6.42e+02	1.84e+03	3.00e+02	3.00e+02	9.16e+03	3.82e+03	9.39e+03	**3.00e+02**	5.70e+04
F4	std	1.27e+00	5.16e-01	2.85e+01	3.34e+00	7.01e+01	1.34e+00	1.05e+00	3.53e+01	4.42e+00	1.00e+00	1.02e+00	**1.33e-01**
F4	mean	4.05e+02	4.07e+02	4.49e+02	4.05e+02	4.53e+02	4.02e+02	4.02e+02	4.34e+02	4.09e+02	4.07e+02	4.02e+02	**4.01e+02**
F5	std	3.37e+00	5.44e+00	1.90e+01	1.76e+01	9.86e+00	1.16e+01	8.19e+00	1.38e+01	1.18e+01	3.40e+00	**2.27e+00**	1.40e+01
F5	mean	**5.06e+02**	5.14e+02	5.57e+02	5.33e+02	5.39e+02	5.20e+02	5.33e+02	5.48e+02	5.21e+02	5.18e+02	5.17e+02	5.24e+02
F6	std	**1.32e-12**	5.14e-02	7.67e+00	8.58e+00	8.07e+00	1.06e+00	2.60e-01	1.20e+01	1.99e+00	2.76e-07	1.26e-08	5.84e-06
F6	mean	**6.00e+02**	6.00e+02	6.22e+02	6.07e+02	6.19e+02	6.01e+02	6.00e+02	6.37e+02	6.02e+02	6.00e+02	6.00e+02	6.00e+02
F7	std	2.68e+00	**1.60e+00**	1.70e+01	8.58e+00	2.19e+01	7.30e+00	1.07e+01	2.28e+01	1.67e+01	3.41e+00	5.45e+00	4.88e+00
F7	mean	7.19e+02	**7.18e+02**	7.60e+02	7.60e+02	7.87e+02	7.41e+02	7.38e+02	7.86e+02	7.34e+02	7.29e+02	7.29e+02	7.38e+02
F8	std	**1.27e+00**	3.55e+00	3.55e+00	4.48e+00	4.62e+00	7.56e+00	5.54e+00	2.02e+01	7.24e+00	2.45e+00	2.64e+00	6.57e+00
F8	mean	**8.07e+02**	8.11e+02	8.33e+02	8.18e+02	8.29e+02	8.18e+02	8.24e+02	8.44e+02	8.17e+02	8.19e+02	8.14e+02	8.25e+02
F9	std	4.00e-02	2.71e-03	1.36e+02	5.08e+01	8.86e+01	6.04e+01	1.06e+01	3.47e+02	3.89e+01	7.24e-07	0.00e+00	**0.00e+00**
F9	mean	9.00e+02	9.00e+02	1.08e+03	9.27e+02	1.02e+03	9.38e+02	9.09e+02	1.63e+03	9.21e+02	9.00e+02	9.00e+02	**9.00e+02**
F10	std	**1.04e+02**	2.52e+02	2.60e+02	5.54e+02	1.69e+02	3.39e+02	1.53e+02	5.02e+02	3.38e+02	1.79e+02	1.52e+02	2.22e+02
F10	mean	**1.17e+03**	1.69e+03	2.22e+03	1.87e+03	1.75e+03	1.69e+03	1.85e+03	2.15e+03	1.97e+03	1.93e+03	1.95e+03	2.67e+03
F11	std	1.91e+00	1.86e+00	1.39e+02	1.31e+01	2.63e+01	4.41e+01	3.37e+01	7.27e+01	2.01e+01	**7.08e-01**	8.54e-01	1.07e+02
F11	mean	**1.10e+03**	1.11e+03	1.26e+03	1.13e+03	1.15e+03	1.14e+03	1.12e+03	1.23e+03	1.14e+03	1.11e+03	1.10e+03	1.25e+03
F13	std	1.06e+01	4.10e+02	5.44e+02	6.14e+03	3.96e+03	8.86e+03	7.58e+02	1.05e+04	1.29e+04	3.37e+03	**4.83e+00**	2.72e+04
F13	mean	1.32e+03	2.02e+03	2.28e+03	1.47e+04	3.76e+03	7.85e+03	2.19e+03	1.40e+04	1.35e+04	4.39e+03	**1.31e+03**	6.13e+04
F14	std	9.89e+00	7.06e+01	2.66e+01	1.40e+03	1.92e+01	8.53e+02	2.69e+01	6.19e+02	1.99e+03	5.95e+01	**8.66e+00**	7.96e+03
F14	mean	**1.41e+03**	1.51e+03	1.50e+03	2.42e+03	1.46e+03	1.93e+03	1.49e+03	2.37e+03	4.39e+03	1.48e+03	1.41e+03	1.29e+04
F15	std	2.82e+00	3.13e+02	7.12e+02	2.19e+03	7.36e+01	1.44e+02	4.74e+01	9.49e+03	6.49e+03	3.50e+02	**4.54e-01**	2.55e+04
F15	mean	1.51e+03	1.90e+03	2.17e+03	5.01e+03	1.62e+03	1.90e+03	1.63e+03	1.24e+04	8.84e+03	1.83e+03	**1.50e+03**	2.77e+04
F16	std	5.80e+01	1.84e+01	1.52e+02	9.25e+01	1.36e+02	1.04e+02	1.40e+02	2.00e+02	9.63e+01	1.15e+01	**3.51e+00**	5.18e+01
F16	mean	1.63e+03	1.62e+03	1.89e+03	1.85e+03	1.83e+03	1.71e+03	1.79e+03	1.89e+03	1.73e+03	1.63e+03	**1.60e+03**	1.68e+03
F17	std	9.85e+00	1.13e+01	2.43e+01	**6.14e+00**	9.36e+00	3.36e+01	3.74e+01	9.81e+01	1.66e+01	7.85e+00	8.29e+00	1.99e+01
F17	mean	**1.71e+03**	1.74e+03	1.80e+03	1.76e+03	1.74e+03	1.76e+03	1.75e+03	1.87e+03	1.77e+03	1.72e+03	1.72e+03	1.80e+03
F18	std	1.03e+01	5.02e+03	2.39e+03	4.43e+03	5.89e+03	1.29e+04	7.96e+03	6.27e+03	1.26e+04	2.09e+03	**8.25e+00**	8.87e+04
F18	mean	1.82e+03	7.67e+03	4.50e+03	9.57e+03	5.55e+03	1.73e+04	8.43e+03	1.31e+04	3.21e+04	4.93e+03	**1.82e+03**	2.02e+05
F19	std	**4.10e-01**	2.15e+03	7.92e+02	4.04e+03	2.34e+01	1.84e+03	7.33e+01	6.98e+04	3.51e+03	4.02e+02	6.56e-01	1.24e+04
F19	mean	1.90e+03	2.98e+03	2.50e+03	7.42e+03	1.93e+03	3.27e+03	2.04e+03	4.77e+04	4.76e+03	2.56e+03	**1.90e+03**	1.87e+04
F20	std	8.98e+00	1.58e+01	1.07e+02	3.88e+01	2.06e+01	1.43e+02	1.11e+02	6.04e+01	6.28e+01	**1.69e-03**	1.05e+01	1.82e+01
F20	mean	2.00e+03	2.02e+03	2.15e+03	2.11e+03	2.07e+03	2.14e+03	2.11e+03	2.21e+03	2.10e+03	**2.00e+03**	2.01e+03	2.09e+03
F21	std	**9.49e-01**	3.99e+00	6.13e+01	3.42e+01	7.50e+01	5.62e+01	7.07e+01	7.77e+01	5.65e+01	2.53e+01	5.33e+01	1.15e+01
F21	mean	**2.20e+03**	2.31e+03	2.32e+03	2.22e+03	2.26e+03	2.30e+03	2.28e+03	2.34e+03	2.30e+03	2.31e+03	2.30e+03	2.33e+03
F22	std	4.51e+01	**4.66e-01**	2.51e+01	1.46e+00	6.44e+00	1.27e+00	9.41e-01	7.19e+00	9.50e+00	6.61e-01	4.73e-01	9.68e+02
F22	mean	**2.28e+03**	2.30e+03	2.37e+03	2.31e+03	2.31e+03	2.30e+03	2.30e+03	2.32e+03	2.32e+03	2.30e+03	2.30e+03	3.01e+03
F23	std	**3.18e+00**	4.28e+00	2.52e+01	6.65e+00	1.74e+01	1.96e+01	1.00e+01	3.22e+01	1.43e+01	3.29e+00	3.45e+00	7.13e+00
F23	mean	**2.61e+03**	2.61e+03	2.65e+03	2.63e+03	2.64e+03	2.63e+03	2.63e+03	2.66e+03	2.63e+03	2.62e+03	2.62e+03	2.64e+03
F24	std	1.28e+00	6.51e+00	1.22e+02	1.19e+02	1.42e+02	1.71e+01	1.20e+01	3.14e+01	2.13e+01	**1.21e+00**	3.73e+00	9.37e+00
F24	mean	2.73e+03	2.75e+03	2.69e+03	2.71e+03	**2.69e+03**	2.76e+03	2.76e+03	2.80e+03	2.75e+03	2.76e+03	2.75e+03	2.74e+03
F25	std	2.49e+01	**4.85e+00**	5.00e+01	2.05e+01	2.29e+01	2.55e+01	2.07e+01	3.70e+01	1.24e+01	1.55e+01	2.50e+01	2.09e+01
F25	mean	2.92e+03	**2.90e+03**	2.97e+03	2.94e+03	2.92e+03	2.93e+03	2.94e+03	2.95e+03	2.94e+03	2.92e+03	2.93e+03	2.94e+03
F26	std	**4.55e-13**	3.37e+01	4.86e+02	1.59e+02	1.85e+02	9.09e+01	3.92e+02	5.28e+02	4.73e+02	3.37e+01	2.48e+01	8.66e+01
F26	mean	**2.90e+03**	2.92e+03	3.30e+03	3.06e+03	3.00e+03	2.94e+03	3.20e+03	3.53e+03	3.60e+03	2.94e+03	2.91e+03	3.31e+03
F27	std	1.71e+00	1.51e+00	4.26e+01	6.83e+00	4.31e+00	2.49e+00	2.79e+01	4.98e+01	1.11e+01	**7.34e-01**	4.17e+00	9.38e-01
F27	mean	3.09e+03	3.09e+03	3.13e+03	3.11e+03	3.10e+03	3.10e+03	3.11e+03	3.15e+03	3.10e+03	3.09e+03	3.09e+03	**3.07e+03**
F28	std	1.10e+02	1.54e+01	1.48e+02	1.44e+02	1.07e+02	2.14e+02	2.32e+02	7.65e+01	8.58e+01	8.45e+01	1.31e+02	**1.22e+01**
F28	mean	3.36e+03	**3.18e+03**	3.47e+03	3.25e+03	3.23e+03	3.43e+03	3.50e+03	3.47e+03	3.38e+03	3.29e+03	3.33e+03	3.28e+03
F29	std	1.12e+01	4.26e+01	5.03e+01	1.23e+01	4.18e+01	5.05e+01	6.91e+01	2.45e+02	4.72e+01	2.38e+01	**9.78e+00**	1.88e+01
F29	mean	**3.16e+03**	3.19e+03	3.27e+03	3.21e+03	3.19e+03	3.26e+03	3.23e+03	3.44e+03	3.21e+03	3.19e+03	3.19e+03	3.30e+03
F30	std	**5.58e+03**	3.91e+05	1.88e+06	5.21e+05	1.01e+04	1.70e+06	4.53e+05	2.21e+06	6.93e+05	1.47e+05	5.84e+05	5.92e+04
F30	mean	**8.00e+03**	5.39e+05	1.54e+06	3.75e+05	1.63e+04	1.68e+06	7.44e+05	2.12e+06	6.31e+05	1.82e+05	4.19e+05	7.35e+04

Note: The best results in each table are highlighted in boldface.

**Table 6:** Comparative results of 12 algorithms on cec2017 test functions (D = 50).

		IPKO	PKO	NRBO	KO	POA	HBA	GBO	WOA	GWO	DE	LSHADE	CMAES
F1	std	2.58e+05	1.00e+08	3.12e+09	5.19e+07	1.58e+10	3.44e+09	7.57e+06	9.43e+09	3.62e+09	2.05e+07	**4.29e+03**	1.07e+05
F1	mean	3.80e+05	2.32e+08	5.21e+10	1.21e+08	4.23e+10	3.28e+09	1.72e+07	2.20e+10	8.17e+09	5.55e+07	**8.62e+03**	1.64e+05
F3	std	2.30e+04	7.32e+04	3.00e+04	1.79e+04	**1.59e+04**	2.53e+04	2.69e+04	1.10e+05	1.98e+04	3.26e+04	3.79e+04	3.40e+05
F3	mean	**1.15e+05**	3.91e+05	1.83e+05	1.56e+05	1.22e+05	1.23e+05	1.26e+05	3.49e+05	1.64e+05	3.77e+05	2.18e+05	9.72e+05
F4	std	4.50e+01	5.30e+01	2.78e+03	8.67e+01	1.37e+03	7.88e+01	3.82e+01	7.40e+02	4.78e+02	3.03e+01	4.88e+01	**4.02e-01**
F4	mean	5.96e+02	6.76e+02	8.73e+03	7.36e+02	9.11e+03	6.84e+02	6.79e+02	4.39e+03	1.59e+03	7.26e+02	6.06e+02	**4.46e+02**
F5	std	5.32e+01	2.11e+01	3.28e+01	2.80e+01	3.03e+01	4.68e+01	3.77e+01	1.42e+02	4.33e+01	1.91e+01	2.84e+01	**1.76e+01**
F5	mean	**7.29e+02**	7.66e+02	1.11e+03	8.62e+02	9.43e+02	7.95e+02	8.18e+02	1.14e+03	7.69e+02	9.38e+02	8.45e+02	8.68e+02
F6	std	1.73e+00	1.28e+01	6.26e+00	1.24e+01	4.22e+00	6.60e+00	4.59e+00	7.48e+00	6.05e+00	3.60e-01	6.33e-01	**6.34e-02**
F6	mean	6.06e+02	6.20e+02	6.96e+02	6.74e+02	6.71e+02	6.37e+02	6.44e+02	6.92e+02	6.24e+02	6.03e+02	6.01e+02	**6.00e+02**
F7	std	4.68e+01	8.55e+01	1.81e+02	1.01e+02	4.68e+01	1.95e+01	9.79e+01	7.72e+01	7.77e+01	**1.27e+01**	2.11e+01	1.85e+01
F7	mean	**1.00e+03**	1.13e+03	1.74e+03	1.33e+03	1.77e+03	1.32e+03	1.24e+03	1.89e+03	1.15e+03	1.20e+03	1.16e+03	1.12e+03
F8	std	1.60e+01	3.32e+01	3.42e+01	2.47e+01	4.57e+01	2.08e+01	3.79e+01	4.38e+01	5.88e+01	**1.11e+01**	3.49e+01	9.07e+01
F8	mean	**1.01e+03**	1.07e+03	1.46e+03	1.15e+03	1.28e+03	1.08e+03	1.09e+03	1.35e+03	1.04e+03	1.25e+03	1.19e+03	1.12e+03
F9	std	2.28e+03	2.46e+03	4.63e+03	6.19e+03	1.66e+03	3.76e+03	3.54e+03	2.11e+03	6.27e+03	2.54e+03	9.04e+02	**3.09e-01**
F9	mean	5.14e+03	5.46e+03	2.74e+04	2.91e+04	1.77e+04	1.29e+04	8.96e+03	3.07e+04	1.63e+04	9.77e+03	1.59e+03	**9.01e+02**
F10	std	8.98e+02	1.04e+03	6.75e+02	2.79e+03	6.09e+02	1.90e+03	1.09e+03	7.71e+02	4.29e+02	2.98e+02	**2.54e+02**	3.03e+02
F10	mean	**7.79e+03**	9.15e+03	1.43e+04	1.15e+04	8.90e+03	1.08e+04	8.25e+03	1.38e+04	8.04e+03	1.43e+04	1.47e+04	1.58e+04
F11	std	6.31e+01	9.75e+02	2.06e+03	4.16e+02	1.42e+03	1.69e+02	7.53e+01	1.37e+03	2.20e+03	2.20e+03	**5.26e+01**	5.82e+04
F11	mean	**1.39e+03**	3.27e+03	8.96e+03	2.29e+03	6.66e+03	1.71e+03	1.55e+03	8.24e+03	7.45e+03	7.65e+03	1.42e+03	1.31e+05
F13	std	**3.62e+03**	3.52e+05	1.92e+09	3.79e+05	4.14e+09	3.42e+04	1.01e+04	2.76e+08	1.17e+08	6.30e+06	3.63e+04	8.90e+06
F13	mean	**4.97e+03**	3.86e+05	4.34e+09	6.78e+05	4.74e+09	6.49e+04	2.81e+04	4.88e+08	1.10e+08	1.05e+07	4.00e+04	1.91e+07
F14	std	1.76e+05	1.72e+05	2.28e+06	1.74e+06	2.13e+06	2.27e+05	1.27e+05	3.93e+06	4.43e+05	1.24e+06	**1.03e+05**	2.21e+06
F14	mean	1.71e+05	7.57e+05	3.15e+06	1.99e+06	1.52e+06	2.57e+05	**1.10e+05**	6.02e+06	1.01e+06	3.10e+06	2.18e+05	4.10e+06
F15	std	**1.47e+03**	1.18e+05	2.59e+08	6.79e+04	2.33e+08	3.22e+04	1.03e+04	6.45e+07	2.80e+08	2.29e+05	2.36e+04	7.24e+06
F15	mean	**4.00e+03**	1.12e+05	4.69e+08	1.23e+05	1.95e+08	5.71e+04	1.57e+04	5.78e+07	2.25e+08	3.54e+05	4.05e+04	1.80e+07
F16	std	3.64e+02	8.48e+02	6.49e+02	4.71e+02	7.45e+02	5.05e+02	4.72e+02	1.53e+03	5.37e+02	**2.36e+02**	3.91e+02	4.63e+02
F16	mean	**3.04e+03**	3.41e+03	6.12e+03	4.21e+03	5.05e+03	3.68e+03	3.98e+03	6.33e+03	3.39e+03	5.02e+03	4.49e+03	4.70e+03
F17	std	2.43e+02	3.82e+02	2.29e+02	4.85e+02	1.82e+02	2.44e+02	2.18e+02	3.91e+02	2.43e+02	**9.01e+01**	1.98e+02	6.33e+02
F17	mean	**2.58e+03**	3.15e+03	4.36e+03	3.76e+03	3.49e+03	3.18e+03	3.22e+03	4.65e+03	3.14e+03	3.81e+03	3.68e+03	4.72e+03
F18	std	**2.30e+05**	2.02e+06	2.01e+07	1.35e+06	7.91e+06	4.43e+05	3.13e+05	2.74e+07	7.73e+06	6.01e+06	1.32e+06	8.17e+06
F18	mean	6.35e+05	3.06e+06	3.19e+07	4.11e+06	4.71e+06	1.21e+06	**4.40e+05**	4.09e+07	6.43e+06	1.13e+07	2.96e+06	1.68e+07
F19	std	8.53e+03	2.77e+04	8.31e+07	4.71e+05	5.25e+06	**7.29e+03**	1.49e+04	8.49e+06	1.01e+07	4.57e+05	1.50e+04	3.31e+06
F19	mean	2.02e+04	4.05e+04	2.54e+08	8.24e+05	6.41e+06	**1.26e+04**	2.35e+04	1.88e+07	9.26e+06	6.02e+05	2.81e+04	8.56e+06
F20	std	2.59e+02	2.51e+02	3.11e+02	5.81e+02	2.86e+02	7.19e+01	2.65e+02	4.96e+02	1.47e+02	1.07e+02	**6.35e+01**	6.77e+01
F20	mean	**2.63e+03**	3.13e+03	3.89e+03	3.80e+03	3.04e+03	3.31e+03	3.10e+03	3.93e+03	2.87e+03	3.75e+03	3.91e+03	4.04e+03
F21	std	3.28e+01	4.88e+01	7.67e+01	7.25e+01	5.45e+01	5.35e+01	8.40e+01	6.81e+01	3.21e+01	2.79e+01	3.17e+01	**1.69e+01**
F21	mean	**2.45e+03**	2.57e+03	3.04e+03	2.65e+03	2.85e+03	2.56e+03	2.58e+03	3.14e+03	2.56e+03	2.73e+03	2.66e+03	2.66e+03
F22	std	1.64e+03	1.26e+03	2.48e+03	3.09e+03	1.03e+03	1.14e+03	3.86e+03	1.47e+03	1.01e+03	**3.08e+02**	3.19e+02	3.65e+02
F22	mean	8.94e+03	1.12e+04	1.50e+04	1.50e+04	1.15e+04	9.27e+03	**8.85e+03**	1.48e+04	9.69e+03	1.57e+04	1.59e+04	1.73e+04
F23	std	3.18e+01	4.05e+01	1.24e+02	1.47e+02	8.99e+01	9.33e+01	9.76e+01	1.77e+02	6.73e+01	**1.07e+01**	5.41e+01	1.08e+02
F23	mean	**2.88e+03**	3.01e+03	3.63e+03	3.28e+03	3.60e+03	3.12e+03	3.16e+03	3.82e+03	3.09e+03	3.18e+03	3.12e+03	3.05e+03
F24	std	3.07e+01	2.60e+01	1.09e+02	8.25e+01	1.93e+02	4.29e+02	1.28e+02	1.03e+02	5.68e+01	**1.36e+01**	1.96e+01	9.72e+01
F24	mean	**3.03e+03**	3.15e+03	3.78e+03	3.41e+03	3.80e+03	3.43e+03	3.28e+03	3.99e+03	3.19e+03	3.35e+03	3.28e+03	3.18e+03
F25	std	4.97e+01	4.99e+01	1.84e+03	8.54e+01	1.50e+03	6.02e+01	2.38e+01	2.91e+02	3.56e+02	7.13e+01	2.80e+01	**6.58e-02**
F25	mean	3.15e+03	3.19e+03	6.87e+03	3.28e+03	7.08e+03	3.25e+03	3.16e+03	5.25e+03	3.58e+03	3.16e+03	3.08e+03	**2.93e+03**
F26	std	2.05e+03	4.91e+02	2.19e+03	3.21e+03	5.72e+02	2.97e+03	2.88e+03	1.55e+03	8.22e+02	**1.49e+02**	2.24e+02	3.50e+02
F26	mean	**6.22e+03**	6.25e+03	1.30e+04	7.36e+03	1.36e+04	7.42e+03	6.34e+03	1.42e+04	6.90e+03	7.95e+03	7.58e+03	7.22e+03
F27	std	7.81e+01	3.00e+01	5.86e+02	1.18e+02	1.60e+02	2.87e+02	3.05e+02	6.36e+02	7.55e+01	6.46e+01	7.65e+01	**4.94e-05**
F27	mean	3.45e+03	3.50e+03	4.75e+03	4.00e+03	4.20e+03	4.32e+03	3.73e+03	4.78e+03	3.62e+03	3.58e+03	3.44e+03	**3.20e+03**
F28	std	2.98e+01	3.90e+02	6.84e+02	1.14e+02	6.39e+02	1.46e+02	8.43e+01	5.11e+02	1.94e+02	5.14e+02	6.74e+01	**3.64e-05**
F28	mean	3.45e+03	3.81e+03	7.30e+03	3.70e+03	6.76e+03	3.74e+03	3.46e+03	5.88e+03	4.72e+03	3.99e+03	3.40e+03	**3.30e+03**
F29	std	3.15e+02	3.37e+02	7.18e+02	2.64e+02	8.86e+02	7.13e+02	3.20e+02	2.41e+03	2.97e+02	2.19e+02	2.96e+02	**1.90e+02**
F29	mean	**4.38e+03**	4.99e+03	8.15e+03	5.27e+03	7.62e+03	5.22e+03	4.92e+03	9.06e+03	4.94e+03	5.54e+03	4.83e+03	5.83e+03
F30	std	**9.14e+04**	1.46e+07	3.73e+08	3.40e+07	8.54e+07	9.79e+05	2.87e+05	6.35e+07	4.56e+07	6.47e+06	9.69e+05	4.09e+06
F30	mean	**9.52e+05**	1.86e+07	7.51e+08	8.60e+07	1.96e+08	3.42e+06	1.33e+06	2.87e+08	1.55e+08	3.10e+07	1.84e+06	1.15e+07

Note: The best results in each table are highlighted in boldface.

**Table 7:** Comparative results of 12 algorithms on cec2017 test functions (D = 100).

		IPKO	PKO	NRBO	KO	POA	HBA	GBO	WOA	GWO	DE	LSHADE	CMAES
F1	std	7.00e+08	2.00e+09	1.19e+10	1.19e+09	1.80e+10	1.09e+10	5.21e+08	1.17e+10	7.51e+09	6.45e+08	6.22e+08	**1.64e+07**
F1	mean	1.28e+09	1.10e+10	1.69e+11	5.42e+09	1.48e+11	4.30e+10	1.79e+09	1.05e+11	5.33e+10	4.77e+09	4.41e+08	**9.40e+07**
F3	std	5.45e+04	2.54e+05	2.53e+04	1.09e+04	2.53e+04	1.12e+05	4.95e+04	7.99e+04	**1.09e+05**	1.00e+05	8.32e+04	8.15e+05
F3	mean	3.38e+05	9.74e+05	3.62e+05	3.56e+05	**3.12e+05**	4.06e+05	3.76e+05	7.87e+05	5.47e+05	8.48e+05	6.09e+05	1.97e+06
F4	std	1.31e+02	3.67e+02	9.61e+03	4.59e+02	4.36e+03	9.95e+02	1.57e+02	2.29e+03	1.46e+03	1.37e+02	6.43e+01	**2.21e+00**
F4	mean	1.14e+03	1.68e+03	3.32e+04	2.01e+03	2.25e+04	2.84e+03	1.53e+03	1.84e+04	7.30e+03	1.81e+03	9.96e+02	**5.05e+02**
F5	std	6.29e+01	1.60e+02	2.62e+01	**2.41e+01**	3.18e+01	5.95e+01	1.07e+02	1.44e+02	4.43e+01	5.45e+01	6.84e+01	3.27e+01
F5	mean	**1.21e+03**	1.33e+03	1.97e+03	1.50e+03	1.62e+03	1.32e+03	1.31e+03	1.99e+03	1.28e+03	1.66e+03	1.46e+03	1.41e+03
F6	std	1.77e+01	5.03e+00	2.35e+00	7.79e+00	4.51e+00	5.63e+00	4.15e+00	9.79e+00	3.33e+00	1.34e+00	3.77e+00	**5.75e-01**
F6	mean	6.40e+02	6.50e+02	7.04e+02	6.88e+02	6.83e+02	6.57e+02	6.61e+02	7.11e+02	6.47e+02	6.22e+02	6.15e+02	**6.04e+02**
F7	std	1.89e+02	2.43e+02	2.54e+02	2.19e+02	1.19e+02	1.51e+02	2.09e+02	1.01e+02	1.27e+02	5.46e+01	6.38e+01	**5.34e+01**
F7	mean	2.33e+03	2.16e+03	3.67e+03	2.56e+03	3.52e+03	2.69e+03	2.71e+03	3.70e+03	2.13e+03	2.14e+03	2.01e+03	**1.72e+03**
F8	std	**1.37e+02**	1.20e+02	1.06e+02	3.06e+01	7.31e+01	7.46e+01	4.59e+01	3.24e+01	3.32e+01	1.14e+01	6.61e+01	2.75e+01
F8	mean	1.48e+03	1.66e+03	2.41e+03	2.00e+03	2.14e+03	1.72e+03	1.76e+03	2.32e+03	1.56e+03	1.93e+03	1.81e+03	**1.74e+03**
F9	std	6.47e+03	1.19e+04	8.02e+03	1.16e+04	5.11e+03	1.48e+04	1.61e+03	1.53e+04	1.23e+04	8.50e+03	5.24e+03	**3.66e+01**
F9	mean	3.27e+04	3.14e+04	7.33e+04	6.97e+04	4.06e+04	5.19e+04	2.96e+04	6.99e+04	5.04e+04	6.07e+04	1.46e+04	**1.08e+03**
F10	std	1.96e+03	4.22e+02	1.15e+03	5.29e+03	8.36e+02	1.82e+03	8.92e+02	1.55e+03	5.78e+03	6.15e+02	**3.26e+02**	6.11e+02
F10	mean	**1.69e+04**	2.33e+04	3.09e+04	2.81e+04	2.17e+04	2.08e+04	1.87e+04	3.02e+04	2.11e+04	3.21e+04	3.19e+04	3.42e+04
F11	std	1.08e+04	4.77e+04	2.83e+04	9.54e+03	1.08e+04	1.18e+04	**7.56e+03**	6.27e+04	1.57e+04	2.12e+04	1.85e+04	4.07e+05
F11	mean	**3.45e+04**	2.73e+05	1.19e+05	9.70e+04	9.14e+04	6.75e+04	3.76e+04	2.52e+05	9.43e+04	2.60e+05	1.41e+05	9.98e+05
F13	std	4.66e+03	1.49e+06	5.30e+09	2.83e+05	5.22e+09	9.57e+04	1.38e+04	1.31e+09	1.82e+09	4.39e+05	**4.50e+03**	7.22e+07
F13	mean	**1.01e+04**	7.91e+05	1.26e+10	3.37e+06	1.20e+10	2.73e+05	9.18e+04	3.11e+09	2.65e+09	5.66e+05	1.42e+04	3.76e+08
F14	std	5.94e+05	5.05e+06	7.19e+06	3.31e+06	6.78e+05	1.54e+06	**3.86e+05**	9.32e+06	5.74e+06	1.54e+07	1.34e+06	7.95e+07
F14	mean	1.79e+06	1.15e+07	1.84e+07	7.53e+06	7.65e+06	2.60e+06	**1.28e+06**	2.07e+07	9.08e+06	4.09e+07	4.91e+06	8.35e+07
F15	std	**4.36e+02**	2.14e+05	9.84e+08	1.54e+05	2.64e+09	1.98e+04	8.17e+03	4.29e+08	5.50e+08	1.26e+06	1.38e+03	1.05e+08
F15	mean	**2.80e+03**	1.46e+05	5.53e+09	3.96e+05	4.31e+09	5.06e+04	2.45e+04	6.56e+08	5.38e+08	8.74e+05	4.17e+03	2.67e+08
F16	std	6.07e+02	6.72e+02	1.19e+03	2.66e+02	1.36e+03	7.99e+02	4.74e+02	9.57e+02	3.33e+02	**2.33e+02**	4.02e+02	3.80e+02
F16	mean	**5.22e+03**	7.34e+03	1.38e+04	8.43e+03	1.16e+04	6.52e+03	6.15e+03	1.62e+04	6.85e+03	1.14e+04	9.50e+03	1.14e+04
F17	std	3.72e+02	4.29e+02	3.84e+04	7.50e+02	4.11e+04	7.89e+02	5.71e+02	1.07e+04	2.02e+03	**2.81e+02**	3.66e+02	1.51e+03
F17	mean	**4.58e+03**	6.44e+03	1.23e+05	5.53e+03	5.52e+04	6.30e+03	5.70e+03	1.83e+04	6.23e+03	7.80e+03	6.87e+03	1.41e+04
F18	std	**1.19e+06**	7.13e+06	1.21e+07	3.06e+06	6.61e+06	1.69e+06	1.79e+06	7.89e+06	7.61e+06	1.53e+07	2.88e+06	3.86e+07
F18	mean	3.23e+06	1.41e+07	3.09e+07	7.13e+06	9.05e+06	4.19e+06	**2.07e+06**	1.67e+07	9.11e+06	7.38e+07	9.77e+06	1.56e+08
F19	std	2.51e+03	5.94e+05	1.14e+09	2.99e+06	2.86e+09	6.17e+05	7.49e+04	4.30e+08	3.93e+08	1.59e+07	**2.20e+03**	1.28e+08
F19	mean	**3.71e+03**	7.98e+05	3.47e+09	4.93e+06	5.51e+09	5.10e+05	6.83e+04	7.57e+08	3.98e+08	1.16e+07	4.10e+03	4.36e+08
F20	std	2.12e+02	2.89e+02	2.76e+02	4.23e+02	**2.02e+02**	1.16e+03	5.80e+02	6.39e+02	1.60e+03	4.48e+02	4.33e+02	2.62e+02
F20	mean	5.28e+03	5.98e+03	7.31e+03	5.59e+03	**5.02e+03**	5.56e+03	5.66e+03	7.12e+03	5.53e+03	7.67e+03	7.57e+03	8.04e+03
F21	std	6.20e+01	1.11e+02	1.39e+02	1.70e+02	4.63e+01	9.30e+01	1.31e+02	1.60e+02	8.25e+01	2.91e+01	**1.79e+01**	3.31e+01
F21	mean	**2.84e+03**	3.21e+03	4.11e+03	3.34e+03	4.05e+03	3.05e+03	3.20e+03	4.29e+03	3.12e+03	3.50e+03	3.34e+03	3.24e+03
F22	std	1.97e+03	2.67e+03	1.54e+03	4.98e+03	1.46e+03	4.22e+03	8.47e+02	1.37e+03	1.54e+03	4.23e+02	**3.61e+02**	1.05e+03
F22	mean	**2.12e+04**	2.37e+04	3.33e+04	3.02e+04	2.38e+04	2.45e+04	2.27e+04	3.12e+04	2.13e+04	3.42e+04	3.42e+04	3.57e+04
F23	std	9.28e+01	1.07e+02	1.89e+02	9.63e+01	5.69e+02	3.81e+02	1.15e+02	1.77e+02	1.05e+02	3.93e+01	1.00e+02	**1.96e+01**
F23	mean	**3.36e+03**	3.69e+03	5.12e+03	4.23e+03	4.83e+03	4.14e+03	3.78e+03	5.28e+03	3.70e+03	3.80e+03	3.78e+03	3.78e+03
F24	std	6.85e+01	7.11e+01	2.90e+02	1.75e+02	5.77e+02	1.44e+03	1.73e+02	7.33e+02	1.17e+02	**1.27e+01**	1.29e+02	2.65e+01
F24	mean	**3.84e+03**	4.17e+03	6.43e+03	4.96e+03	6.33e+03	5.68e+03	4.47e+03	6.68e+03	4.49e+03	4.35e+03	4.39e+03	4.19e+03
F25	std	**6.71e+01**	6.67e+02	8.94e+02	2.29e+02	1.39e+03	4.37e+02	9.48e+01	9.64e+02	1.04e+03	5.41e+02	7.20e+01	6.74e+01
F25	mean	3.88e+03	5.19e+03	1.51e+04	4.85e+03	1.26e+04	5.56e+03	4.14e+03	1.02e+04	7.76e+03	5.83e+03	3.61e+03	**3.35e+03**
F26	std	2.03e+03	1.53e+03	2.22e+03	2.55e+03	1.80e+03	2.50e+03	2.52e+03	3.88e+03	1.93e+03	3.82e+02	8.95e+02	**1.94e+02**
F26	mean	**1.41e+04**	1.48e+04	3.51e+04	2.47e+04	3.63e+04	1.89e+04	1.88e+04	3.94e+04	1.82e+04	1.72e+04	1.69e+04	1.52e+04
F27	std	6.64e+01	1.24e+02	5.17e+02	5.09e+02	8.05e+02	6.39e+02	1.98e+02	1.40e+03	1.49e+02	1.46e+02	4.05e+01	**5.25e-05**
F27	mean	3.75e+03	3.66e+03	6.19e+03	4.71e+03	6.19e+03	4.20e+03	3.84e+03	6.65e+03	4.15e+03	4.43e+03	3.62e+03	**3.20e+03**
F28	std	2.02e+02	1.25e+03	1.41e+03	6.49e+02	3.04e+03	9.49e+02	3.03e+02	1.71e+03	1.08e+03	1.01e+03	2.30e+02	**3.36e-05**
F28	mean	4.22e+03	6.95e+03	1.92e+04	5.65e+03	1.74e+04	6.74e+03	4.63e+03	1.52e+04	8.96e+03	1.10e+04	4.21e+03	**3.30e+03**
F29	std	9.06e+02	5.71e+02	1.18e+04	1.02e+03	1.24e+03	6.53e+02	7.40e+02	3.22e+03	5.21e+02	4.40e+02	**4.16e+02**	2.07e+03
F29	mean	**7.54e+03**	9.07e+03	2.95e+04	1.10e+04	1.65e+04	8.40e+03	8.04e+03	2.05e+04	9.30e+03	1.03e+04	9.64e+03	1.84e+04
F30	std	1.46e+05	7.79e+06	3.19e+09	2.86e+07	2.63e+09	1.38e+07	4.30e+06	1.24e+09	1.38e+09	5.31e+06	**4.16e+04**	6.60e+07
F30	mean	3.00e+05	8.64e+06	1.20e+10	9.42e+07	1.30e+10	1.62e+07	3.75e+06	3.30e+09	1.52e+09	1.52e+07	**7.34e+04**	5.65e+08

Note: The best results in each table are highlighted in boldface.

**Table 8:** Friedman test results for the CEC2017 benchmark functions at 10 dimensions.

Algorithms	MeanRank	Ranking	Source	SS	DF	MS	Chi-sq	Prob > Chi-sq
IPKO	**2.25**	**1**	-	-	-	-	-	-
LSHADE	3.09	2	-	-	-	-	-	-
PKO	4.25	3	-	-	-	-	-	-
DE	4.86	4	-	-	-	-	-	-
GBO	6.39	5	-	-	-	-	-	-
POA	6.46	6	-	-	-	-	-	-
HBA	6.89	7	-	-	-	-	-	-
KO	7.25	8	-	-	-	-	-	-
CMAES	8.05	9	-	-	-	-	-	-
GWO	8.14	10	-	-	-	-	-	-
NRBO	9.32	11	-	-	-	-	-	-
WOA	11.04	12	-	-	-	-	-	-
Friedman’s ANOVA			Columns	692.79	9	76.9762	75.58	$1.6034\times 10^{-27}$
			Error	1617.21	243	6.6552		
			Total	2310	279			

*Note*: MeanRank denotes the average rank derived from the Friedman test; ranking is sorted in ascending order (a smaller value indicates better algorithm performance); the *p*-value ($1.6034\times 10^{-27}<0.05$) verifies significant statistical differences among the compared algorithms. The best results in each table are highlighted in boldface.

**Table 9:** Friedman test results for the CEC2017 benchmark functions at 30 dimensions.

Algorithms	MeanRank	Ranking	Source	SS	DF	MS	Chi-sq	Prob > Chi-sq
IPKO	**1.93**	**1**	-	-	-	-	-	-
LSHADE	4.14	2	-	-	-	-	-	-
GBO	4.59	3	-	-	-	-	-	-
PKO	4.76	4	-	-	-	-	-	-
HBA	5.45	5	-	-	-	-	-	-
GMAES	6.07	6	-	-	-	-	-	-
GWO	6.28	7	-	-	-	-	-	-
KO	7	8	-	-	-	-	-	-
DE	7.31	9	-	-	-	-	-	-
POA	8.86	10	-	-	-	-	-	-
NRBO	10.34	11	-	-	-	-	-	-
WOA	11.28	12	-	-	-	-	-	-
Friedman’s ANOVA			Columns	637.74	9	70.86	69.57	$9.6894\times 10^{-32}$
			Error	1754.76	252	6.96		
			Total	2392.5	289			

*Note*: MeanRank denotes the average rank derived from the Friedman test; ranking is sorted in ascending order (a smaller value indicates better algorithm performance); the *p*-value ($9.6894\times 10^{-32}<0.05$) verifies significant statistical differences among the compared algorithms. The best results in each table are highlighted in boldface.

**Table 10:** Friedman test results for the CEC2017 benchmark functions at 50 dimensions.

Algorithms	MeanRank	Ranking	Source	SS	DF	MS	Chi-sq	Prob > Chi-sq
IPKO	**1.64**	**1**	-	-	-	-	-	-
GBO	4.11	2	-	-	-	-	-	-
PKO	4.61	3	-	-	-	-	-	-
LSHADE	4.86	4	-	-	-	-	-	-
HBA	5.25	5	-	-	-	-	-	-
GWO	5.82	6	-	-	-	-	-	-
CMAES	6.54	7	-	-	-	-	-	-
KO	7.25	8	-	-	-	-	-	-
DE	7.54	9	-	-	-	-	-	-
POA	8.86	10	-	-	-	-	-	-
NRBO	10.71	11	-	-	-	-	-	-
WOA	10.82	12	-	-	-	-	-	-
Friedman’s ANOVA			Columns	642.43	9	71.381	70.08	$1.1017\times 10^{-31}$
			Error	1667.57	243	6.8624		
			Total	2310	279			

*Note*: MeanRank denotes the average rank derived from the Friedman test; ranking is sorted in ascending order (a smaller value indicates better algorithm performance); the *p*-value ($1.1017\times 10^{-31}<0.05$) verifies significant statistical differences among the compared algorithms. The best results in each table are highlighted in boldface.

**Table 11:** Friedman test results for the CEC2017 benchmark functions at 100 dimensions.

Algorithms	MeanRank	Ranking	Source	SS	DF	MS	Chi-sq	Prob > Chi-sq
IPKO	**2**	**1**	-	-	-	-	-	-
GBO	3.93	2	-	-	-	-	-	-
LSHDE	4.93	3	-	-	-	-	-	-
HBA	5.39	4	-	-	-	-	-	-
PKO	5.43	5	-	-	-	-	-	-
GWO	5.75	6	-	-	-	-	-	-
CMAES	6.43	7	-	-	-	-	-	-
KO	6.71	8	-	-	-	-	-	-
DE	7.75	9	-	-	-	-	-	-
POA	8.68	10	-	-	-	-	-	-
WOA	10.43	11	-	-	-	-	-	-
NRBO	10.57	12	-	-	-	-	-	-
Friedman’s ANOVA			Columns	561.36	9	62.373	61.24	$5.4369\times 10^{-27}$
			Error	1748.64	243	7.1961		
			Total	2310	279			

*Note*: MeanRank denotes the average rank derived from the Friedman test; ranking is sorted in ascending order (a smaller value indicates better algorithm performance); the *p*-value ($5.4369\times 10^{-27}<0.05$) verifies significant statistical differences among the compared algorithms. The best results in each table are highlighted in boldface.

**Table 12:** Holm post-hoc test results comparing IPKO with other algorithms (D = 10).

Compare	IPKO Rank	Other Rank	Raw *p*-Value	Holm *p*-Value	Significance (α=0.05)
PKO	2.25	4.25	3.79e-02	7.59e-02	No
NRBO	2.25	9.32	2.16e-13	2.16e-12	Yes
KO	2.25	7.25	2.12e-07	1.48e-06	Yes
POA	2.25	6.46	1.22e-05	6.12e-05	Yes
HBA	2.25	6.89	1.45e-06	8.70e-06	Yes
GBO	2.25	6.39	1.71e-05	6.86e-05	Yes
WOA	2.25	11.04	<$1\times 10^{-15}$	<$1\times 10^{-15}$	Yes
GWO	2.25	8.14	9.64e-10	8.67e-09	Yes
DE	2.25	4.86	6.82e-03	2.05e-02	Yes
LSHADE	2.25	3.09	3.84e-01	3.84e-01	No
CMAES	2.25	8.05	1.72e-09	1.37e-08	Yes

**Table 13:** Holm post-hoc test results comparing IPKO with other algorithms (D = 30).

Compare	IPKO Rank	Other Rank	Raw *p*-Value	Holm *p*-Value	Significance (α=0.05)
PKO	1.93	4.76	2.82e-03	8.47e-03	Yes
NRBO	1.93	10.34	<$1\times 10^{-15}$	<$1\times 10^{-15}$	Yes
KO	1.93	7	8.63e-08	6.04e-07	Yes
POA	1.93	8.86	2.48e-13	2.23e-12	Yes
HBA	1.93	5.45	2.04e-04	8.14e-04	Yes
GBO	1.93	4.59	5.04e-03	1.01e-02	Yes
WOA	1.93	11.28	<$1\times 10^{-15}$	<$1\times 10^{-15}$	Yes
GWO	1.93	6.28	4.46e-06	2.68e-05	Yes
DE	1.93	7.31	1.34e-08	1.07e-07	Yes
LSHADE	1.93	4.14	1.98e-02	1.98e-02	Yes
CMAES	1.93	6.07	1.24e-05	6.21e-05	Yes

**Table 14:** Holm post-hoc test results comparing IPKO with other algorithms (D = 50).

Compare	IPKO Rank	Other Rank	Raw *p*-Value	Holm *p*-Value	Significance (α=0.05)
PKO	1.64	4.61	2.10e-03	4.19e-03	Yes
NRBO	1.64	10.71	<$1\times 10^{-15}$	<$1\times 10^{-15}$	Yes
KO	1.64	7.25	5.93e-09	4.15e-08	Yes
POA	1.64	8.86	7.06e-14	6.35e-13	Yes
HBA	1.64	5.25	1.82e-04	7.26e-04	Yes
GBO	1.64	4.11	1.05e-02	1.05e-02	Yes
WOA	1.64	10.82	<$1\times 10^{-15}$	<$1\times 10^{-15}$	Yes
GWO	1.64	5.82	1.45e-05	7.24e-05	Yes
DE	1.64	7.54	9.64e-10	7.71e-09	Yes
LSHADE	1.64	4.86	8.51e-04	2.55e-03	Yes
CMAES	1.64	6.54	3.82e-07	2.29e-06	Yes

**Table 15:** Holm post-hoc test results comparing IPKO with other algorithms (D = 100).

Compare	IPKO Rank	Other Rank	Raw *p*-Value	Holm *p*-Value	Significance (α=0.05)
PKO	2	5.43	3.74e-04	1.49e-03	Yes
NRBO	2	10.57	<$1\times 10^{-15}$	<$1\times 10^{-15}$	Yes
KO	2	6.71	9.97e-07	6.98e-06	Yes
POA	2	8.68	4.19e-12	3.77e-11	Yes
HBA	2	5.39	4.30e-04	1.29e-03	Yes
GBO	2	3.93	4.54e-02	4.54e-02	Yes
WOA	2	10.43	<$1\times 10^{-15}$	<$1\times 10^{-15}$	Yes
GWO	2	5.75	9.96e-05	4.98e-04	Yes
DE	2	7.75	2.42e-09	1.93e-08	Yes
LSHADE	2	4.93	2.37e-03	4.75e-03	Yes
CMAES	2	6.43	4.31e-06	2.59e-05	Yes

**Table 16:** Overall mean CPU runtime (seconds) ± standard deviation across 30 CEC2017 functions (D = 30).

Algorithm	Mean Runtime (s)	Std Runtime (s)
IPKO	0.768	0.666
PKO	0.358	0.257
NRBO	0.219	0.152
KO	1.031	0.864
POA	0.281	0.185
HBA	0.188	0.130
GBO	0.265	0.180
WOA	**0.115**	0.082
GWO	0.197	0.142
DE	0.258	0.191
LSHADE	0.186	0.125
CMAES	0.709	0.668

Note: The best results in each table are highlighted in boldface.

**Table 17:** Average performance ranking of algorithms under different FEs budgets across dimensions.

Algorithm	30D (5K/10K/15K)	50D (5K/10K/15K)	100D (5K/10K/15K)	Overall Trend
IPKO	**1.7**/**1.6**/**1.7**	**2.3**/**1.6**/**1.7**	**2.5**/**2.0**/**1.9**	Consistently best
GBO	3.1/3.8/4.7	2.5/3.0/3.8	3.5/3.5/4.1	Very competitive, often 2nd
LSHADE	3.8/4.0/4.0	4.0/4.5/5.1	3.5/4.3/4.8	Stable top-3 performer
HBA	4.7/4.3/5.2	5.2/4.8/5.2	6.3/5.0/5.8	Mid-range, degrades on higher dim
CMAES	6.5/6.4/6.1	6.5/6.5/6.5	6.0/6.2/6.4	Robust, but not top
PKO	6.7/5.1/4.8	6.5/4.9/4.9	7.3/5.9/5.5	Improves with more evaluations
DE	7.0/7.0/7.0	7.0/7.3/7.4	7.5/7.3/7.6	Consistent middle rank
GWO	7.8/7.9/6.4	7.6/7.8/5.9	6.6/7.3/6.0	Better on high budget
POA	7.9/8.4/8.6	8.1/8.5/8.8	8.4/8.6/8.7	Poor overall
KO	8.6/8.4/7.8	8.3/8.1/7.1	7.8/7.7/6.6	Improves slightly with budget
NRBO	9.4/10.2/10.5	9.8/10.5/10.8	9.5/10.3/10.6	Among worst
WOA	10.7/11.1/11.2	10.1/10.6/10.9	9.2/9.9/10.2	Consistently worst or near-worst

Note: The best results in each table are highlighted in boldface.

**Table 18:** Geometric mean of final errors and Budget Efficiency Ratio (BER) for representative algorithms under 30D and 100D.

Algorithm	30D			100D		
	Error (5 k)	Error (15k)	BER (%)	Error (5k)	Error (15k)	BER (%)
IPKO	**6.42e+02**	**2.15e+02**	66.5	**2.34e+04**	**3.45e+03**	85.3
GBO	1.13e+04	1.03e+03	90.9	1.52e+05	2.94e+04	80.7
LSHADE	4.56e+03	3.76e+02	91.8	1.06e+05	2.25e+04	78.8
WOA	1.59e+06	1.12e+06	29.6	2.48e+06	1.95e+06	21.4

Note: The best results in each table are highlighted in boldface.

**Table 19:** Friedman rankings for different population sizes (c=2.0).

Pop	20	25	30	35	40	45	50	55	60	65	70	75	80
Rank	6.13	**3.00**	3.75	4.38	5.25	7.25	6.50	8.13	7.75	8.63	9.88	9.50	10.88

Note: The best results in each table are highlighted in boldface.

**Table 20:** Friedman rankings for different *c* values (Pop=30).

*c*	0.5	1.0	1.5	2.0	2.5	3.0
Rank	3.88	3.63	4.00	3.25	**2.50**	3.75

Note: The best results in each table are highlighted in boldface.

**Table 21:** Mean fitness values for different (Pop, *c*) combinations across eight CEC2017 functions. Lower values indicate better performance.

c\Pop	20	25	30	35	40	45	50	55	60	65	70	75	80
0.5	3.3285	3.3151	3.3349	3.3324	3.3802	3.4157	3.4776	3.5145	3.5803	3.6264	3.6346	3.6743	3.7236
1.0	3.3413	3.3130	3.2947	3.3440	3.3609	3.4251	3.4532	3.5373	3.5636	3.6077	3.6532	3.6937	3.7143
1.5	3.3201	3.3296	3.3029	3.2866	3.3936	3.4209	3.4317	3.4828	3.5414	3.6069	3.6365	3.6544	3.6991
2.0	3.3541	**3.2587**	3.2862	3.3167	3.3773	3.4082	3.4401	3.4995	3.5466	3.5787	3.6371	3.6588	3.7018
2.5	3.3780	3.2773	3.2691	3.3012	3.3908	3.3974	3.4538	3.4941	3.5570	3.5820	3.6540	3.6876	3.6939
3.0	3.3392	3.2916	3.2989	3.3316	3.3815	3.3915	3.4431	3.4800	3.5276	3.5881	3.6427	3.7017	3.7158

Note: The best results in each table are highlighted in boldface.

**Table 22:** Ablation study results on CEC2017 functions (30D, 30 runs).

Function	Metric	IPKO	PKO	IPKOa	IPKOab	IPKOabc
F3	Mean	**2.14e+04**	1.60e+05	1.47e+05	4.81e+04	2.20e+04
	Std	5.75e+03	4.83e+04	2.70e+04	8.59e+03	4.82e+03
	Rank	1	5	4	3	2
F4	Mean	**4.88e+02**	5.20e+02	5.16e+02	5.22e+02	5.01e+02
	Std	2.27e+01	1.85e+01	1.88e+01	1.31e+01	1.87e+01
	Rank	1	4	3	5	2
F5	Mean	**5.72e+02**	6.02e+02	5.92e+02	6.23e+02	5.86e+02
	Std	1.66e+01	2.83e+01	2.61e+01	3.32e+01	1.66e+01
	Rank	1	4	3	5	2
F8	Mean	**8.69e+02**	8.97e+02	8.93e+02	9.10e+02	8.81e+02
	Std	1.57e+01	2.72e+01	2.35e+01	2.13e+01	1.86e+01
	Rank	1	4	3	5	2
F9	Mean	**1.10e+03**	1.41e+03	1.40e+03	1.90e+03	1.39e+03
	Std	3.78e+02	4.71e+02	6.60e+02	1.03e+03	5.43e+02
	Rank	1	4	3	5	2
F10	Mean	**4.19e+03**	5.07e+03	5.19e+03	5.08e+03	4.54e+03
	Std	5.79e+02	6.21e+02	6.34e+02	5.57e+02	5.63e+02
	Rank	1	3	5	4	2
F14	Mean	**3.96e+03**	8.73e+04	8.73e+04	6.56e+04	6.54e+03
	Std	3.14e+03	6.72e+04	5.43e+04	6.03e+04	8.89e+03
	Rank	1	4	5	3	2
F18	Mean	**8.26e+04**	1.07e+06	1.48e+06	4.06e+05	1.28e+05
	Std	5.31e+04	1.11e+06	1.90e+06	2.78e+05	1.34e+05
	Rank	1	4	5	3	2
F24	Mean	**2.87e+03**	2.91e+03	2.91e+03	2.90e+03	2.88e+03
	Std	1.47e+01	2.25e+01	2.43e+01	2.67e+01	1.44e+01
	Rank	1	5	4	3	2
F28	Mean	**3.23e+03**	3.29e+03	3.30e+03	3.28e+03	3.24e+03
	Std	1.86e+01	2.50e+01	4.22e+01	2.25e+01	2.12e+01
	Rank	1	4	5	3	2
**Average rank**		**1.00**	4.03	4.20	3.70	2.00

Note: The best results in each row are marked in bold.

**Table 23:** Customer demand point data.

ID	*x* (km)	*y* (km)	Demand (t)	Soft Start (h)	Soft End (h)	Hard Start (h)	Hard End (h)
1	12	32	0.5	4.83	5.83	4.33	6.33
2	18	35	0.4	5.00	6.00	4.50	6.50
3	10	37	0.8	5.33	6.33	4.83	6.83
4	20	30	0.4	5.67	6.67	5.17	7.17
5	17	26	0.6	6.83	7.83	6.33	8.33
6	22	23	0.8	5.50	6.50	5.00	7.00
7	20	36	0.4	6.00	7.00	5.50	7.50
8	13	21	0.7	6.00	7.00	5.50	7.50
9	23	18	0.9	5.83	6.83	5.33	7.33
10	23	32	0.5	5.17	6.17	4.67	6.67
11	25	27	0.9	5.50	6.50	5.00	7.00
12	26	31	0.3	6.83	7.83	6.33	8.33
13	11	17	1.0	6.17	7.17	5.67	7.67
14	10	25	1.2	5.67	6.67	5.17	7.17
15	9	30	0.9	5.33	6.33	4.83	6.83

**Table 24:** Experimental parameters for cold chain logistics.

Parameter	Meaning	Value	Parameter	Meaning	Value
$p_{1}$	Fixed cost per vehicle (yuan/vehicle)	200	$Q_{1}$	Vehicle self weight (t)	5.3
$p_{2}$	Transportation cost per km (yuan/km)	2	$Q_{2}$	Rated load capacity (t)	5
$p_{3}$	Unit price of fresh products (yuan/t)	3000	$e_{1}$	Transport carbon factor (kg/L)	2.63
$p_{5}$	Carbon price (yuan/kg)	0.2	$e_{2}$	Refrigeration carbon factor (kg/L)	2.63
$p_{\mathrm{fuel}}$	Diesel price (yuan/L)	7.5	SFC	Refrigeration SFC (L/kWh)	0.28
${\mathrm{arf}}_{1}$	Transport loss rate	0.01	$\rho_{1}$	Empty fuel consumption (L/km)	0.165
${\mathrm{arf}}_{2}$	Door-opening loss rate	0.15	$\rho_{2}$	Full fuel consumption (L/km)	0.233
*v*	Vehicle speed (km/h)	45	$Z_{\max}$	Max load (t)	5
$t_{0}$	Departure time (h)	5	$L_{\max}$	Max mileage (km)	180
ϕ	Degradation degree	0.08	ω	Unloading efficiency (t/h)	4
*R*	Heat transfer coefficient	2	*u*	Door opening frequency	0.5
$S_{w}$	Outer surface area (m^2^)	75	*V*	Carriage volume (m^3^)	15
$S_{n}$	Inner surface area (m^2^)	50	$T_{n}$	Inside temperature (°C)	0
$T_{w}$	Outside temperature (°C)	25			

**Table 25:** Statistical results of total cost on the cold chain logistics problem over 30 independent runs.

Algorithm	Mean	Std	Best	Worst	Friedman Rank
IPKO	**3984.12**	**21.79**	3943.21	4042.20	**1.67**
PKO	4036.65	45.92	3958.31	4129.74	2.67
PSO	4135.89	71.39	4005.56	4271.94	3.90
GWO	4001.60	44.11	**3912.37**	4109.56	1.90
GA	4344.59	129.41	4104.87	4625.82	4.87
Friedman test p<0.001					

Note: The best results in each table are highlighted in boldface.

**Table 26:** Holm post-hoc test results comparing IPKO with other algorithms (significance level α=0.05).

Comparison	Adjusted *p*-Value	Holm Threshold	Significance
IPKO vs. GA	$1.734\times 10^{-6}$	0.0125	Yes
IPKO vs. PSO	$1.921\times 10^{-6}$	0.0167	Yes
IPKO vs. PKO	$6.320\times 10^{-5}$	0.0250	Yes
IPKO vs. GWO	$1.359\times 10^{-1}$	0.0500	No

**Table 27:** Statistical results on the UAV path planning.

Algorithm	Mean Fitness	Std Fitness	Average Rank (Friedman)
IPKO	**49.24**	3.10	1.73
PKO	52.09	2.89	2.57
IVY	52.21	3.92	2.57
HO	57.10	5.39	4.10
WOA	58.67	6.22	4.03
Friedman test $p=2.13\times 10^{-10}$ (significant at α=0.05).			

Note: The best results in each table are highlighted in boldface.

**Table 28:** Holm post-hoc test results comparing IPKO with other algorithms.

Compare	IPKO Rank	Other Rank	Raw *p*-Value	Holm *p*-Value	Significance (α=0.05)
HO	1.73	4.10	$6.75\times 10^{-9}$	$2.70\times 10^{-8}$	Significant
WOA	1.73	4.03	$1.76\times 10^{-8}$	$5.28\times 10^{-8}$	Significant
IVY	1.73	2.57	$4.12\times 10^{-2}$	$8.25\times 10^{-2}$	Not significant
PKO	1.73	2.57	$4.12\times 10^{-2}$	$4.12\times 10^{-2}$	Not significant

## Data Availability

The data used to create Table 23 (customer coordinates, demand, and time windows) are derived from the case study in Zhu (2020) [44], an unpublished bachelor’s thesis from Changshu Institute of Technology. Further inquiries about this dataset can be directed to the author of that thesis or to Changshu Institute of Technology library.
